# Evaluation of Antibacterial and Cytotoxicity Properties of Silver Nanowires and Their Composites with Carbon Nanotubes for Biomedical Applications

**DOI:** 10.3390/ijms21072303

**Published:** 2020-03-26

**Authors:** Arianna De Mori, Richard S. Jones, Matteo Cretella, Guido Cerri, Roger R. Draheim, Eugen Barbu, Gianluca Tozzi, Marta Roldo

**Affiliations:** 1 School of Pharmacy and Biomedical Science, University of Portsmouth, St Michael’s Building, White Swan Road, PO1 2DT, Portsmouth, UK; arianna.demori@port.ac.uk (A.D.M.); richard.jones1@port.ac.uk (R.S.J.); matteo.cretella@port.ac.uk (M.C.); roger.draheim@port.ac.uk (R.R.D.); eugen.barbu@port.ac.uk (E.B.); 2 Department of Architecture, Design and Urban Planning—GeoMaterials Lab, University of Sassari, Via Piandanna 4, 07100 Sassari, Italy; gcerri@uniss.it; 3 Zeiss Global Centre, School of Engineering, University of Portsmouth, Anglesea Building, Anglesea Road, PO1 3DJ Portsmouth, UK; gianluca.tozzi@port.ac.uk

**Keywords:** silver nanowires, nanomaterials, biocompatibility

## Abstract

In this work, we prepared silver nanowires (AgNWs) via the polyol method in the presence or absence of single wall carbon nanotubes (CNTs) and tested their physicochemical, antibacterial and cytotoxic properties. Results showed that the introduction of CNTs lead to the formation of AgNWs at lower temperature, but the final product characteristics of AgNWs and AgNWs-CNT were not significantly different. AgNWs exhibited antibacterial properties against all the studied bacterial species via the formation of oxygen reactive species (ROS) and membrane damage. Furthermore, AgNWs exhibited a dose-dependent and time-dependent toxicity at concentrations ≥ 10 µg/mL. Fibroblasts appeared to be more resistant than human colorectal adenocarcinoma (Caco-2) and osteoblasts to the toxicity of AgNWs. The cytotoxicity of AgNWs was found to be related to the formation of ROS, but not to membrane damage. Overall, these results suggest that AgNWs are potential antibacterial agents against *E. coli*, *S. aureus*, MRSA and *S. saprophyticus*, but their dosage needs to be adjusted according to the route of administration.

## 1. Introduction

Silver has been known for centuries for its antimicrobial properties against a wide range of microorganisms. In the 1980s the first findings on the unique properties of nanoparticles (NPs) were published, researchers started to investigate novel applications for silver nanomaterials in electronic, optical and biomedical fields. In order to satisfy specific needs, several morphologies have been developed, including silver nanospheres, nanocubes, nanorods, nanotriangles and nanowires.

Silver nanowires (AgNWs) are one-dimensional silver structures and are currently being intensively studied for thermal and electronic applications due to their excellent conductive properties [1,2,3]. Despite the fact that the antibacterial and cytotoxic properties of other silver nanoparticles have been widely explored [4,5,6,7,8] and medical devices loaded with silver nanopoarticles have been developed, research on AgNW antibacterial properties, mechanisms of action and possible biomedical applications is still scarce [9]. So far we know that AgNWs are potential antibacterial agents against *E. coli* and *S. aureus*, but we know little about how they exert their action. Visnapuu et al., for instance, stated that the toxicity of AgNWs against *E. coli* was due to dissolved Ag^+^ ions rather than to a direct effect of the silver nanostructures [10]. Cui and Liu, instead, highlighted that *E. coli* is more sensitive than *S. aureus* to the action of AgNWs, determining their toxicity was a consequence of both AgNW-induced bacterial disruption and the induction of ROS generation [11]. 

In the development of AgNWs containing commercial products, we must be considerate of the fact that nanowires can be released into the environment during manufacturing, use or disposal; thus, there is a compelling need to understand the short- and long-term toxicity of these materials in humans [12]. Other high-aspect ratio materials, such as asbestos or carbon nanotubes, have indeed been shown to be actively absorbed into eukaryotic cells, causing different types of damage [13]. As the respiratory route is one of the major access routes linked to occupational exposure to AgNWs, the majority of studies have so far focused on the possible damages through this route. Schinwald et al. found that AgNWs longer than ≥14 μm or ≥ 5 μm were able to induce pleural inflammation, in vivo and in vitro, respectively [14]. Further studies have also highlighted that AgNWs can enter and accumulate in epithelial cells, interstitial sites, airway smooth muscle cells, the vascular endothelium, the pleural membrane and macrophages. Stoehr et al. compared the cytotoxicity of AgNWs (length: 1.5–25 µm; diameter 100–160 nm) and Ag nanospheres (30 nm) against human alveolar epithelial cells (A549), finding that whereas no effects were observed for the spherical particles, significantly reduced cell viability and increased LDH release were induced by AgNWs [15]. A recent review on the safety of nanosilver has highlighted that, at the nanoscale, silver nanowires are safer than silver nanoparticles due to their hindered cellular uptake [16].

In light of this diverse evidence, and in particular, the results considering different routes of administration, this study aimed to investigate the in vitro antibacterial properties of AgNWs obtained via two synthetic methods (soft template and heterogeneous method) tested against *E. coli, S. aureus,* MRSA and *S. saprophyticus*. Moreover, the internalization, cytotoxicity and possible mechanisms of action of AgNWs against human osteoblasts (hFOB 1.19), human skin fibroblasts (Hs27) and human colorectal adenocarcinoma (Caco-2) were studied in order to assess future potential applications such as inclusion in bone implants or in wound dressings for skin and mucosa. 

## 2. Results

### 2.1. AgNW Physicochemical Characterisation

#### 2.1.1. UV-vis and TEM Characterization

**Figure 1:**
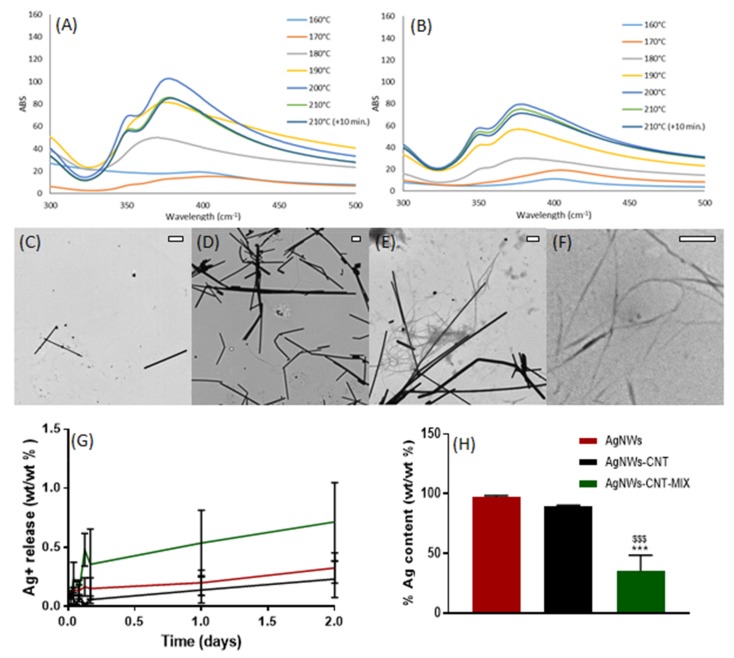
Physicochemical characterization of synthesized products. UV-spectra of (**A**) AgNWs and (**B**) of AgNWs-CNT at different temperatures during the synthesis reaction. TEM images of (**C**) AgNWs (scale bar 500 nm), (**D**) AgNWs-CNT (scale bar 500 nm), (**E**) AgNWs-CNT-mix (scale bar 500 nm) and (**F**) CNTs (scale bar 500 nm). (**G**) Silver release from AgNWs, AgNWs-CNT and AgNWs-CNT-mix measured by ICP-OES and expressed as wt/wt%. (**H**) Silver contents measured by ICP-OES in AgNWs, AgNWs-CNT and AgNWs-CNT-mix (*n* = 3). One-way ANOVA returned *p* < 0.05; Post-hoc Tukey’s multicomparison test, *** *p* = 0.001 when comparing all the formulations to AgNWs, and ^$$$^ *p* = 0.003 when comparing AgNWs-CNT to AgNWs-CNT-mix.

AgNWs were synthesized via the polyol method using PVP as a soft template, and AgNWs-CNT were synthesized with the same method in the presence of carbon nanotubes (CNTs) in order to evaluate whether the addition of CNTs influenced the morphological, physical and biological properties of the AgNWs. AgNWs-CNT-mix (physical mixture of AgNWs and CNTs) was used as a control to investigate whether any potential difference between AgNWs in AgNWs-CNT was due to the mere presence of CNTs or if they had an effect when introduced in the synthetic procedure. The UV-Vis spectra of AgNWs and AgNWs-CNT at low temperatures showed a peak at 410 nm, indicative of the formation of nucleation sites with the initial presence of silver nanoparticles and nanorods (Figure 1A–B). A shift towards lower wavenumbers and the formation of a double peak (350 and 380 nm) were observed as the temperature increased, indicating the formation of longer structures such as nanowires [17]. The shift was observed at lower temperatures when the synthesis of AgNWs was performed in the presence of CNTs; this could be due to the excellent heat conductivity of CNTs that can affect the reaction kinetics. Nanoparticles were also observed by TEM (Figure 1C–F); images confirmed the successful formation of AgNWs under both synthetic conditions. However, in both cases, other types of nanoparticles, such as nanospheres and nanorods, were visualized. Moreover, the amount of CNTs found in AgNWs-CNT appeared significantly inferior to that in the AgNWs-CNT-mix. Interestingly, in both AgNWs-CNT and AgNWs-CNT-mix samples, the smaller particles tended to align along the surface of CNTs (Figure 1G–H). A size analysis was performed based on SEM images (Figures S1 and S2); average lengths were 5.23 μm (± 1.5), 5.21 μm (± 2.7) and 5.04 μm (± 1.7), for AgNWs, AgNWs-CNT and AgNWs-CNT-mix, respectively. Average diameters were 73.70 nm (± 25.79), 67.04 nm (± 25.23) and 68.54 nm (± 17.47) for AgNWs, AgNWs-CNT and AgNWs-CNT-mix, respectively. One-way ANOVA showed no significant difference in lengths and diameters across all three materials (*p* > 0.05).

#### 2.1.2. Total Silver Content and Ag^+^ Release 

The total Ag content in each of the products and the cumulative Ag^+^ released over time were determined. The total silver content found in AgNWs (97.33 ± 1.14 wt/wt%) was higher than that in AgNWs-CNT (89.11 ± 1.25 wt/wt%), but not significantly different (one-way ANOVA, *p*>0.05, Figure 1H); this explains the observation of only a few CNTs in the TEM images (Figure 1C–E). The amount of Ag in AgNWs-CNT-mix was significantly inferior to the other two batches (35.27 ± 5.33 wt/wt%) (*p* < 0.001), as expected. The release of Ag^+^ from AgNWs, AgNWs-CNT and AgNWs-CNT-mix (Figure 1G) started immediately in the aqueous solution and its concentration increased over time. After 30 min, the concentration of Ag^+^ in suspension was ca. 0.31 ± 0.04 ppm from AgNWs, 0.22 ± 0.17 ppm from AgNWs-CNT and 0.09 ± 0.02 ppm from AgNWs-CNT-mix. After 2 days, the percentage of Ag^+^ released was of ca. 0.32 wt/wt% from AgNWs, 0.23 wt/wt% from AgNWs-CNT and 0.71 wt/wt% from AgNWs-CNT-mix (one-way ANOVA *p* > 0.05), corresponding to 1.15 ± 0.39, 0.95 ± 0.53 and 0.92 ± 0.81 ppm, respectively.

#### 2.1.3. XRD, Photoluminescence and ζ-Potential Analyses

**Figure 2:**
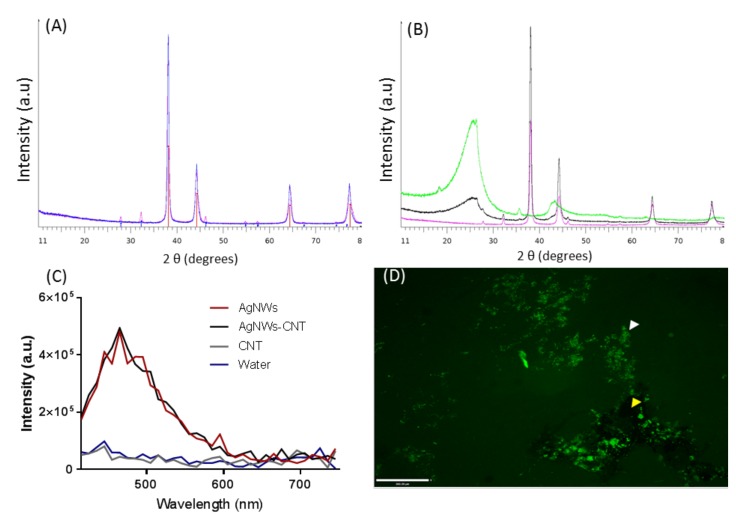
XRD pattern and fluorescence spectra. (**A**) XRD pattern of AgNWs (pink) and AgNWs-CNT (blue); red bars: Ag, PDF No. 04-0783; blue bars: AgCl, PDF n. 31-1238. (**B**) XRD pattern of AgNWs (violet), AgNWs-CNT-mix (black) and CNTs (green). (**C**) The emission fluorescence peaks of AgNWs (red), AgNWs-CNT-mix (black), CNTs (grey) and water (blue). (**D**) Fluorescence microscope image of AgNWs-CNT in water; the white arrow indicates AgNWs, and the yellow arrow indicates CNTs (scale bar 200 µm).

The X-ray diffraction (XRD) pattern of AgNWs (Figure 2A) corresponds to the structure of metallic silver; in particular, the sharp peaks at 2θ values of 38.1°, 44.3°, 64.4° and 77.3° refer to the reticular planes (111), (200), (220) and (311), and are typical of AgNWs [18]. Furthermore, the (111)/(200) peak ratio was 3.7, indicating the formation of well-elongated AgNWs [19]. Minimal trace impurity of AgCl can be seen at a 2θ value of 32.1° and 46.2° [20].

Superimposed patterns of AgNWs and AgNWs-CNT confirmed that the two synthetic processes led to similar products. The (111)/(200) intensity ratio of AgNWs-CNT was 3.2. The XRD profile of CNTs (Figure 2B) showed broad peaks at 2θ angles of ≈26°, ≈43° and ≈57°, corresponding to the (002) plane of graphite and to the (111) and (200) reflections of carbon, respectively [21]; these peaks also featured in AgNWs-CNTs-mix. The (111)/(200) intensity ratio of AgNWs-CNT-mix was found to be 3.2, similar to AgNWs-CNT. The fluorescence emission of AgNWs, AgNWs-CNT and CNTs aqueous solutions was also investigated. Excitation at wavelengths between 390 and 400 nm led to fluorescence emission between 450 and 600 nm with lambda maxima at 465 nm, corresponding to the blue-green emitting region. The luminescence emission from silver clusters and silver nanoparticles of different dimensions has previously been described [22,23], but not entirely understood. This phenomenon could be related to the number and position of surface plasmon resonance (SPR), which in turn depends on the size and shape of silver clusters.

Surface zeta potential (ζ) was found to be negative for all the preparations (AgNWs -12.43 ± 1.23 mV; AgNWs-CNT -18.24 ± 5.92 mV; AgNWs-CNT-mix -19.8 ± 7.66 mV); the values indicated that electrostatically stable suspensions cannot be obtained in water without the use of a suspending agent [10].

### 2.2. Antibacterial Properties

#### 2.2.1. Minimum Inhibitory Concentration (MIC) and Minimum Bactericidal Concentration (MBC)

The potential bacteriostatic and bactericidal activities of the synthesized materials were tested against four different bacterial species: *E. coli*, *S. aureus*, MRSA and *S. saprophyticus.* In order to compare the activity of AgNWs, AgNWs-CNT, AgNWs-CNT-mix and ionic silver (as AgNO_3_), the MIC and MBC values obtained were normalized to the amount of Ag present in each material.

All studied materials inhibited or stopped cell growth within the tested concentration range, except for single-wall carbon nanotubes-COOH (tested as control), which did not show any antibacterial response (Table 1). All preparations were less efficient than AgNO_3_ in both inhibiting cell growth and in promoting cell death apart from *S. saprophyticus* that was highly sensitive to all treatments. Normalized MIC and MBC values of AgNWs, AgNWs-CNT and AgNWs-CNT-mix were not statistically different from each other (*p* > 0.05) against all the different bacterial species; only *S. aureus* AgNWs-CNT-mix showed a lower efficacy (*p* < 0.001). Overall, *S. saprophyticus* was found to be the most sensitive strain against silver nanowires, whereas MRSA was the most resistant. The MIC values found in this study for *E. coli* and *S. aureus* were higher than those reported by Cui and Liu for AgNWs (28 and 35 µg/mL to *E. coli* and *S. aureus*, respectively) [11] and Hong et al. (> 100 µg/mL against *E. coli*) [24]; this can be explained considering the differences in initial bacterial density, nanowire dimensions and methods adopted for the MIC and MBC determination [25].

#### 2.2.2. Bacterial Growth Kinetics

**Figure 3:**
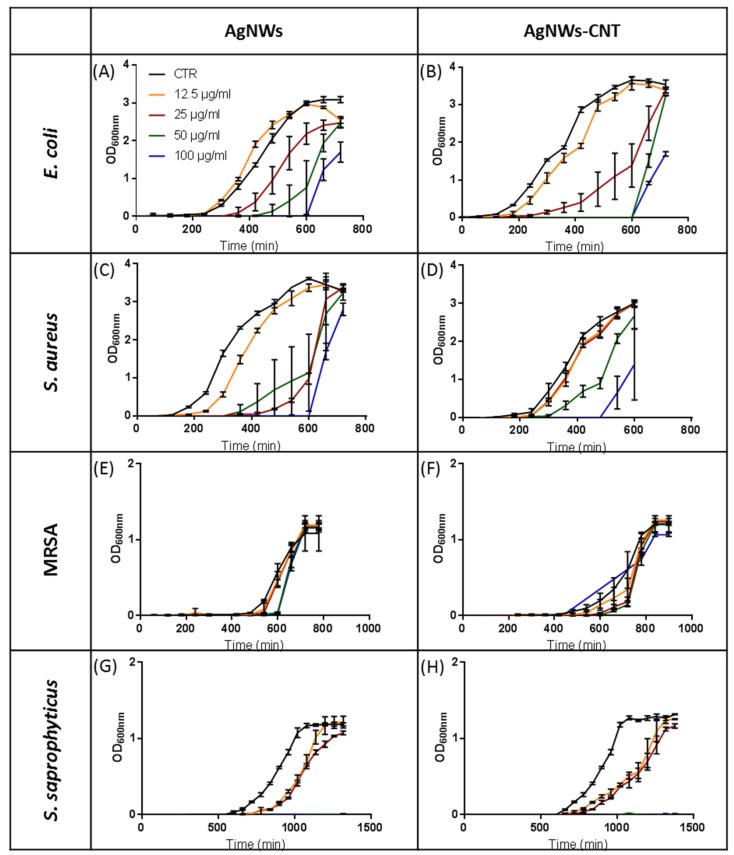
Growth curves of bacteria with different concentrations of AgNWs (**A**,**C**,**E**,**G**) and AgNWs-CNT (**B**,**D**,**F**,**H**). Results are reported as the mean ± SD (*n* = 3).

In order to study how the bacterial growth kinetics were influenced by different concentrations of studied materials, growth was followed by measuring OD._600nm_ in LB medium (Figure 3, Figures S3 and S4). Seemingly for MIC and MBC studies, CNTs did not appear to influence bacterial growth kinetics (Figure S3). AgNWs affected both the lag phase (100 µg/mL, *p* < 0.05) and the time to reach the stationary phase (50 µg/mL, *p* < 0.05) of *E. coli* (Figure 3A and Figure S5). MRSA showed a significantly longer lag phase than the control only in the presence of AgNWs 100 µg/mL (*p* < 0.01) but no effect on the time to reach the stationary phase (Figure 3E and Figure S5). *S. aureus* (Figure 3C) was more sensitive than MRSA and *E. coli* to the action of AgNWs: all the tested concentrations, except for 12.5 µg/mL, produced longer lag phases (25 µg/mL *p* < 0.001; 50 and 100 µg/mL *p* < 0.0001). The time to reach the stationary phase was statistically longer for concentrations equal to or higher than 50 µg/mL (*p <* 0.05). The lag phase duration of *S. saprophyticus* (Figure 3G and Figure S5) was increased for the two lowest concentrations (12.5 µg/mL *p* < 0.01 and 25 µg/mL *p* < 0.05), whereas no growth was found for the higher concentrations of nanowires, and the time to reach the stationary phase was significantly longer even for the lowest tested concentration. *E. coli* was more sensitive to AgNWs-CNT (Figure 3A and Figure S5) than AgNWs: its lag phase (12.5 µg/mL, *p* < 0.05, whereas 25, 50 and 100 µg/mL had a *p* < 0.0001) and the time to reach stationary phase were also significantly longer for the lowest concentration. Seemingly, *S. aureus* (Figure 3D) was more sensitive to the action of AgNWs-CNT than AgNWs with decreased growth at all concentrations tested and no growth at the highest concentration. No difference in the time to reach the stationary phase was found for concentrations 12.5 and 25 µg/mL in comparison to the control (Figure S5). MRSA (Figure 3F) growth was affected by AgNWs-CNT at all concentrations. No effect on the time to reach the stationary phase was observed. *S. saprophyticus* lag phase (Figure 3H and Figure S5) was affected for the two lowest concentrations (*p* < 0.05), and no growth was observed for the higher concentrations. The time to reach the stationary phase of *S. saprophyticus* was statistically longer for the 25 µg/mL concentration than for the control. For all the studied nanowires and bacterial strains, whenever the stationary phase was reached, no statistical difference in OD values was found among treated and untreated bacteria (Figure S5). The ability of AgNWs to retard cell growth was further demonstrated by fluorescence imaging (Figure S6).

#### 2.2.3. Leakage of Proteins From Bacteria

**Figure 4:**
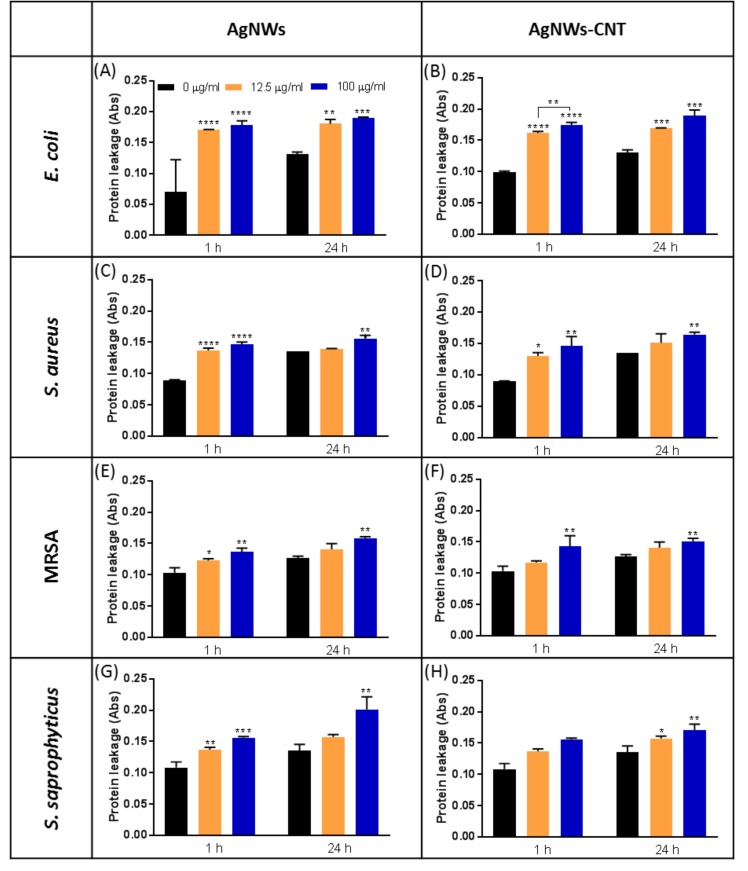
Absorbance relative to protein release after 1 and 24 h of treatment for *E. coli* (**A**,**B**), *S. aureus* (**C**,**D**), MRSA (**E**,**F**) and *S. saprophyticus* (**G**,**H**) treated with 12.5 or 100 µg/mL of AgNWs or AgNWs-CNT. Data are presented as the mean ± SD (*n* = 4). The one-way ANOVA performed on all the samples showed significantly different releases for both AgNWs and AgNWs-CNT (*p* < 0.05). Results of the post-hoc Tukey multicomparison test are shown in the graphs (* indicates *p* < 0.05, ** indicates *p* < 0.01, *** indicates *p* < 0.001 and **** indicates *p* < 0.0001).

Cui and Liu reported that AgNWs could cause the leakage of cytoplasmic contents, such as DNA and proteins, from *E. coli* [11]. This phenomenon may be due to several alterations in bacterial cells, such as the inhibition of the activity of membranous enzymes or DNA damage. In the present study, a bicinchoninic acid (BCA) assay was used to determine whether AgNWs obtained by the two methods induced the release of proteins in the extracellular liquid. All the bacterial species tested showed an increased release of proteins in comparison to the control (Figure 4A–H). Moreover, this effect was dose-dependent, but no difference was found between 1 or 24 h after treatment, except for *S. saprophyticus*. A higher amount of proteins leaked out of *E. coli* compared to *S. aureus* and MRSA, suggesting that gram-negative bacteria are more sensitive than gram-positive bacteria to the action of AgNWs on the integrity of the membrane. This is justified by the barrier function of the thicker peptidoglycan found in gram-positive bacteria and is in agreement with previous studies [26]. No statistical difference was found between AgNWs and AgNWs-CNT, when comparing the same concentrations. SEM images confirmed the cell membrane damage caused by AgNWs, particularly in *E. coli* (Figure S8).

#### 2.2.4. ROS Generation From Bacteria

**Figure 5:**
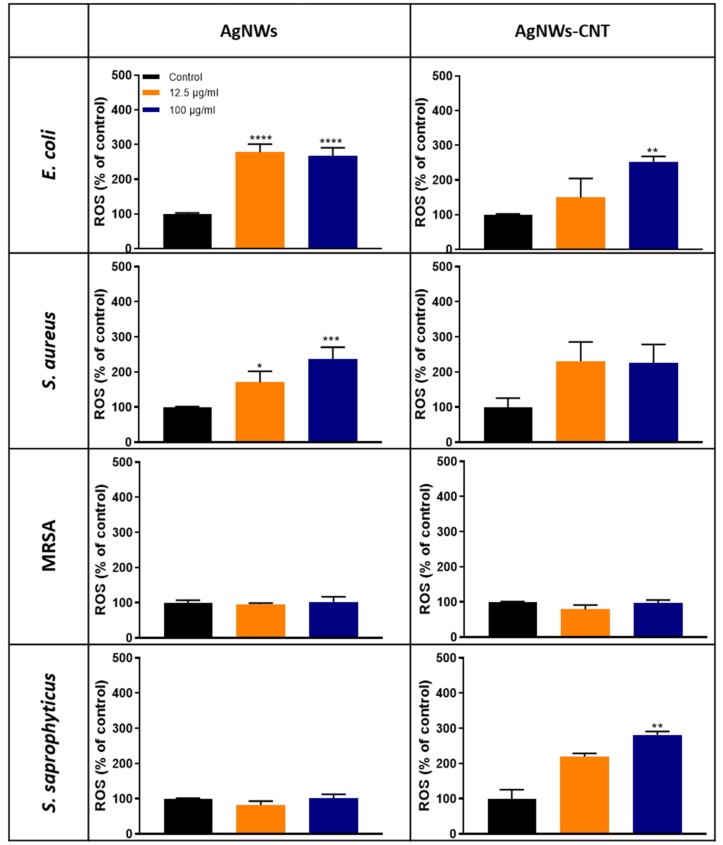
ROS production (% of the control) from bacterial cells, 24 h from treatment. *E. coli* (**A**,**B**), *S. aureus* (**C**,**D**), MRSA (**E**,**F**) and *S. saprophyticus* (**G**,**H**) treated with 12.5 and 100 µg/mL of AgNWs or AgNWs-CNT. Data are presented as the mean ± SD (*n* = 3). The one-way ANOVA performed on all the samples showed significantly different ROS production for both AgNWs and AgNWs-CNT (*p* < 0.05) for some of the bacterial strains. Results of the post-hoc Tukey multicomparison test are shown in the graphs (* indicates *p* < 0.05, ** indicates *p* < 0.01, *** indicates *p* < 0.001 and **** indicates *p* < 0.0001).

Previous studies suggested that oxidative stress plays a crucial role in the mechanism of toxicity of silver nanoparticles versus bacterial cells. In this work, extracellular ROS production was quantified 24 h after treatment with nanowires (Figure 5A–H). ROS were produced when *S. aureus* and *E. coli* were treated with all types of metallic nanoparticles in a dose-dependent manner, as previously reported for other types of AgNPs [27].

MRSA did not show significantly increased ROS generation, similar to *S. saprophyticus* treated with AgNWs; but when *S. saprophyticus* was treated with AgNWs-CNT, there was a statistically significant increase in ROS at concentrations equal to or higher to 25 µg/mL. On the contrary, AgNWs were found to produce statistically more ROS than AgNWs-CNT in *E. coli* and *S. aureus* (unpaired two tailed *t*-test for: a) *E. coli*: 100 *p <* 0.05; 12.5 µg/mL, *p <* 0.01; b) *S. aureus:* 100 µg/mL, *p <* 0.05). See Figure S7 for data obtained for all concentrations tested.

### 2.3. Cytotoxicity Studies

**Figure 6:**
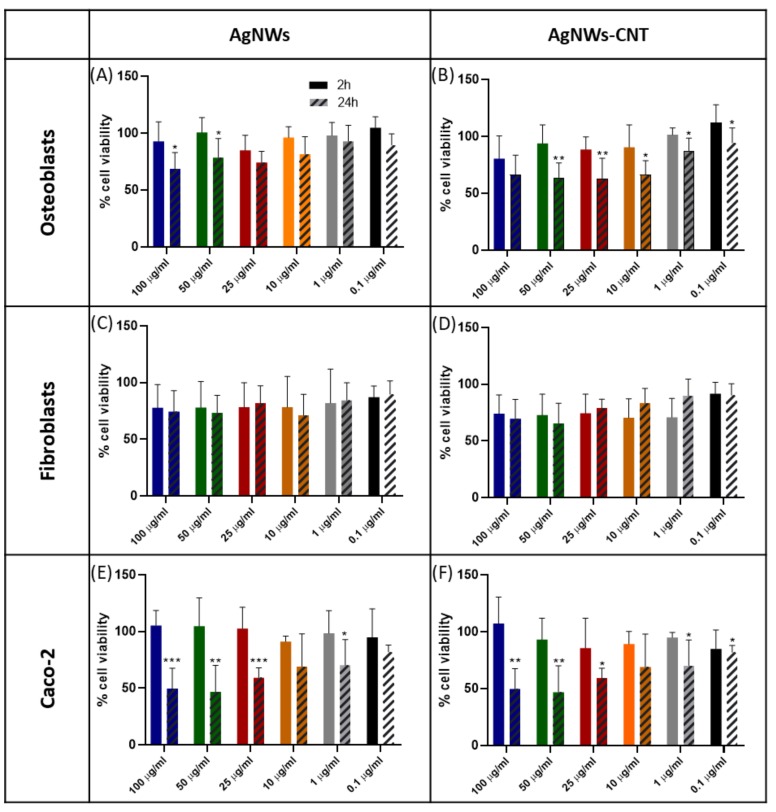
Cytotoxicity observed in different types of cell lines after 2 and 24 h of treatment. Osteoblasts (**A**,**B**), fibroblasts (**C**,**D**), Caco-2 (**E**,**F**). Data are presented as the mean ± SD (*n* > 3). The one-way ANOVA performed on all samples showed significantly different viability for AgNWs and AgNWs-CNT (* indicates *p* < 0.05, ** indicates *p* < 0.01, *** indicates *p* < 0.001 and **** indicates *p* < 0.0001).

The toxicity of the two types of AgNWs was tested against three cell lines: osteoblasts (hFOB 1.19), human foreskin fibroblasts (Hs27) and Caco-2 cells (Figure 6A–F). These cell lines were chosen to mimic three possible routes of exposure such as the application of treatments to bone, skin and the digestive system, respectively [28]. No signs of acute toxicity were observed, as after 2 h of treatment, no cell line showed a significant decrease in cell viability (*p* > 0.05). After 24 h of exposure, fibroblasts were the more robust cells as their viability did not show a significant decrease with increasing doses of AgNWs and AgNWs-CNTs. Caco-2 cells were the most sensitive, with a significant decrease in viability at doses of 25 µg/mL of AgNWs (*p* < 0.001) and 10 µg/mL of AgNWs-CNTs (*p* < 0.001).

#### 2.3.1. Oxidative Stress in Eukaryotic Cells

**Figure 7:**
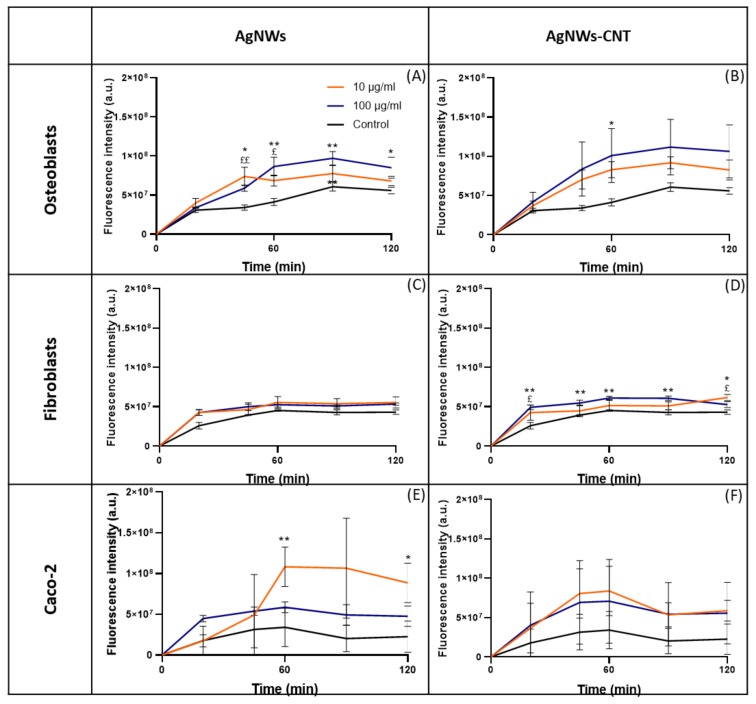
Time course of ROS production in different cell lines: osteoblasts (**A**,**B**), fibroblasts (**C**,**D**) and Caco-2 (**E**,**F**) treated with AgNWs or AgNWs-CNT for 15 min. One-way ANOVA returned *p* < 0.05. Data are reported as the mean ± SD (*n* = 3). Results of the post-hoc Tukey multicomparison test are shown in the graph: *was used to compare 100 with 0 µg/mL (* indicates *p* < 0.05 and ** indicates *p* < 0.01); ^$^ was used to compare 10 with 0 µg/mL (^$^ indicates *p* < 0.05 and ^$$^ indicates *p* < 0.01).

Several studies have shown that AgNPs induce the production of ROS in mammalian cells [29,30]. Results of the present study (Figure 7A–F) confirmed that ROS induced by AgNWs and AgNWs-CNT are an important factor in promoting toxicity, but once again, the effect is specific to the cell line treated. Fibroblasts presented the lowest level of ROS produced when treated with AgNWs-CNTs and with no significant effect when treated with AgNWs. Osteoblasts were the most sensitive to the AgNW treatment, with a higher level of ROS detected. Caco-2 cells presented a wider range of variability that caused non-significant differences when compared to the control. CNTs were tested as a control and induced no significant ROS production in any of the cell lines (Figure S9). Few other studies are available for AgNWs; Sweeney et al. reported that AgNWs (72 nm × 1.5 µm) could induce a significant increase in ROS production in human type-I epithelial-like cells (TT1), both after 4 (≥25 µg/mL) and 24 h treatment (≥ 10 µg/mL) [31].

#### 2.3.2. Membrane Damage Evaluation

**Figure 8:**
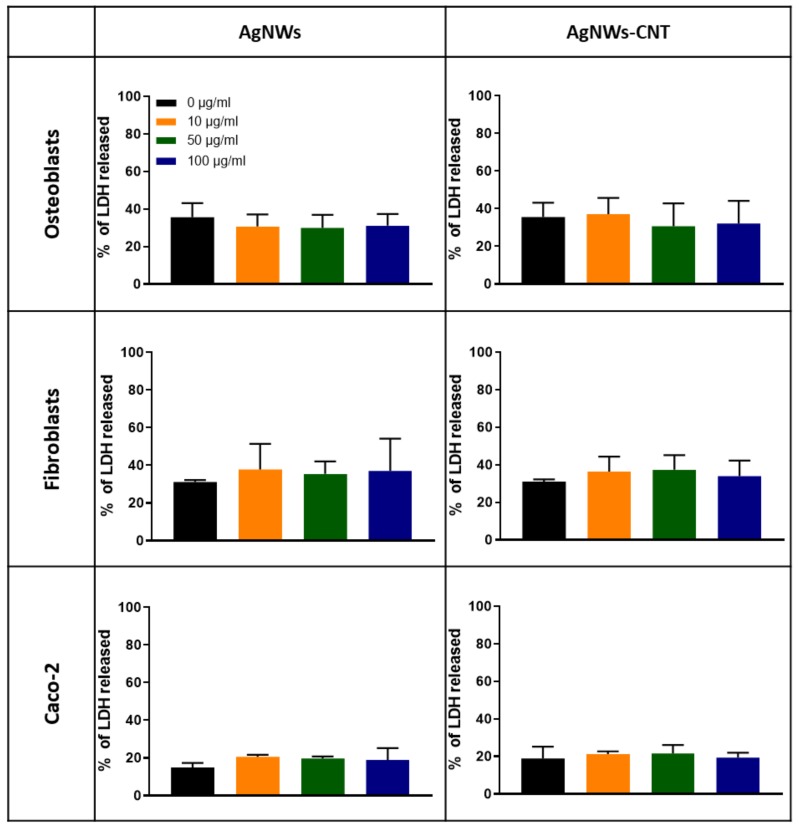
Percentage LDH release from osteoblasts, fibroblasts and Caco-2 after 24 h of exposure to different concentrations of AgNWs or AgNWs-CNTs. The one-way ANOVA calculated among the different concentrations of each test did not show statistical differences (*p* > 0.05). Data are reported as the mean ± SD (*n* = 3).

Membrane integrity was assessed by measuring extracellular lactate dehydrogenase (LDH, Figure 8). No significant toxicity was observed after 24 h for any of the concentrations tested in comparison to the spontaneous LDH release (0 µg/mL) (*p* > 0.05).

#### 2.3.3. Silver Uptake

**Figure 9:**
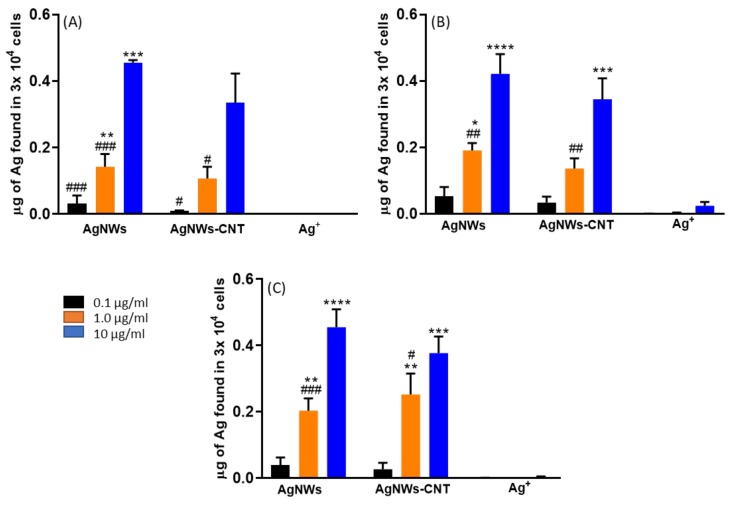
Uptake of silver by (**A**) osteoblasts, (**B**) fibroblasts and (**C**) Caco-2 treated with AgNWs, AgNWs-CNT and AgNO_3_. Data are expressed as the mean ± SD (*n* = 3). One way Anova indicated at least *p* < 0.05 for all samples tested apart from AgNO_3_. Results of the post-hoc Tukey multicomparison test are shown in the graph (symbol *compared to 0.1 and # compared to 10 µg/mL).

Previous studies have reported that AgNWs are taken up and accumulate in different types of lung cells, such as alveolar type-I and type-II epithelial cells [31]. In our work, we wanted to find out if AgNWs are also taken up in other cell lines and at which concentration. Cells were treated with Ag^+^, AgNWs or AgNWs-CNT for 24 h. Concentrations up to 10 µg/mL were tested as they caused no relevant differences in toxicity to osteoblasts after 24 h of exposure (Figure 9).

**Figure 10:**
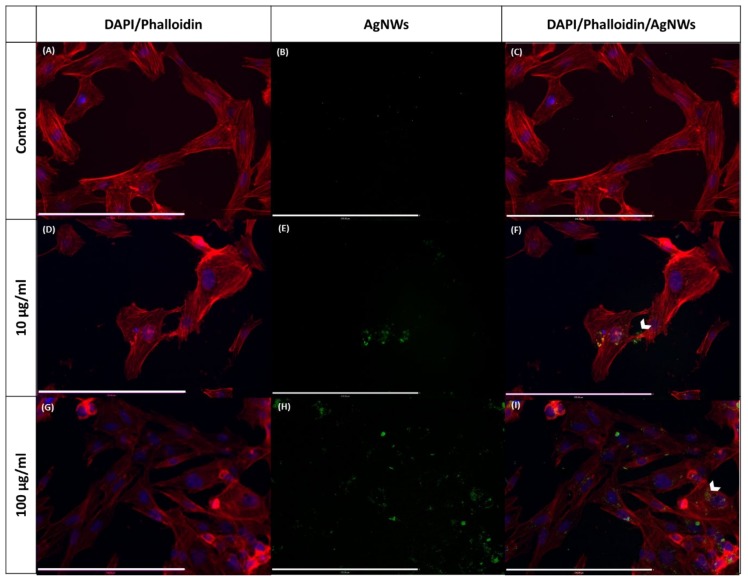
Phalloidin Dylight 550- and DAPI-stained osteoblast cells at 24 h of incubation with AgNWs (10 and 100 µg/mL). (**A**,**D**) and (**G**): merged images of Phalloidin (staining F-actin) and DAPI (staining nuclei) of osteoblasts. (**B**,**E**) and (**H**): AgNWs excited at 495 nm and emitting at 519 nm. (**C**,**F**) and (**I**): merged images of Phalloidin/DAPI/AgNWs. Scale bar 270 µm.

AgNWs were up taken by all cell lines more efficiently than Ag^+^ (Tukey’s multicomparison test showed *p* < 0.0001) and in a concentration-dependent manner. A similar behavior was found with mammalian kidney cells (Pk15) for silver nanoparticles of different sizes (ca. 13.8–61.2 µm) by Milic et al. [32]. We hypothesized that as there is no specific transporter for Ag^+^, it has to compete for transporters with other ionic species, and the amount of Ag that entered in the cells was inferior in comparison to AgNWs that can enter by endocytosis. Results of ICP-OES were confirmed by fluorescence microscopy for osteoblasts (Figure 10), where AgNWs accumulated either close to the membrane (Figure 10F) or within the cytoplasm (Figure 10F–I).

## 3. Discussion

The present study aimed to investigate both the antibacterial efficacy and the eukaryotic cytocompatibility of silver nanowires in vitro, as well as to test the effect of silver nanowire composite materials in combination with carbon nanotubes (based on previous reports of enhanced activity of silver nanowires combined with graphene) [11]. In this work, AgNWs-CNT-mix (a physical mixture of AgNWs and CNTs) was used as a control to further investigate the role of CNTs in the synthesis and properties of the composite material. Silver nanowires were successfully synthesized both in the presence and in absence of carbon nanotubes using the soft template method, as confirmed by UV-VIS, XRD and EM imaging. The average size (ca. 70 nm in diameter and 5 µm in length) and silver content in both materials were similar. In the soft template synthesis of AgNWs, a coordination complex is formed between silver ions and PVP through donation of lone-pair electrons of oxygen and nitrogen atoms of PVP to sp orbitals of the metallic silver reduced by glycerol [33]. We hypothesized that –COOH groups of CNTs can interact with Ag^+^, forming the first nucleation centers for the growth of silver nanowires. TEM images supported the theory that nucleation centers form on the surface of carbon nanotubes and lead to the formation of nanowires along the axis of CNTs. Furthermore, size and silver content data suggest that whereas CNTs might have played a role in the nucleation and reduction of heat required in the synthesis (as seen on UV-Vis spectra), they are not chemically bound to AgNWs, and as a consequence, they are removed during the purification process. The release of Ag^+^ from the nanowires was also studied; the silver ion concentration was higher than 0.1 ppb (minimum effective concentration [34]) already after 30 min, suggesting that these nanomaterials are potential platforms for Ag^+^ controlled release for antibacterial purposes. In fact, even though AgNO_3_ is a more potent antibacterial agent than AgNWs, free silver ions may not act in the long term, as they can be easily washed away by physiological fluids, whereas silver nanowires can act as reservoirs and allow for a sustained release of therapeutic doses [35].

As MIC and MBC values appeared to be similar among AgNWs, AgNWs-CNT and AgNWs-CNT-mix, we could assume that the three preparations had similar antibacterial properties (with no effect due to the presence of CNTs). Moreover, as reported by Cavassin et al., in order to reduce the selection of resistant microorganisms, antimicrobials should present an MBC/MIC ratio ≤4; this was found to be the case with AgNWs against all bacteria tested, apart from *S. aureus* for which the value was 4.3 [36]. In our study, we showed that AgNWs had a bactericidal effect against all the isolates, except for MRSA. The antibacterial activity of AgNWs and AgNWs-CNTs was tested in suspension, and the growth kinetics of all the bacterial strains tested were affected by the treatment but in different ways. Similar results were observed by Cui et Liu, who described the influence of increasing concentrations of AgNWs on *E. coli* growth [11]. However, whereas they showed growth inhibition at concentrations higher than 25 µg/mL, in the present study, none of the concentrations used could inhibit cell growth in the same time frame. Variations in the results can be due to microbial strains, but also to physicochemical characteristics of the AgNW suspension, for instance, the presence of PVP [37]. In our study, MRSA was found to be the least sensitive, whereas *S. saprophyticus* was the most susceptible. These results indicate that it is not possible to explain the species sensitivity to silver nanoparticles in terms of bacterial classification (gram-positive and gram-negative), but that there are more specific differences among species [37]. MRSA has been previously described as susceptible to AgNPs, with evident membrane damage shown by TEM images; the damage was attributed to charge interaction between positively charged nanoparticles and negatively charged peptidoglycan, and only the smaller particles (dia. < 20 nm) were observed to enter the cells [38]. In the case of AgNWs, the negative charge and longer length might hinder the interaction with MRSA and reduce efficacy. In this regard, we previously observed that the combination of AgNWs with the positively charged polymer chitosan had a synergistic effect on AgNW efficacy against MRSA [39]. The mechanism of bacterial toxicity was further investigated with a focus on membrane damage and ROS production. The bacterial toxicity of AgNWs was mainly mediated through oxidative stress, in the case of *E. coli* and *S. aureus*, whereas the ROS levels in MRSA were not significantly changed at the tested concentrations, supporting the theory that MRSA could present more efficient antioxidant mechanisms than *E. coli* and *S. aureus*. In this regard, Grinholc et al. found that MRSA was more resistant than *S. aureus* to photodynamic inactivation, a therapeutic means based on the generation of ROS. The exact reason for this difference was not identified, but evidence led to excluding that it was related to the multidrug resistance mechanisms developed by MRSA [40]. When tested on human cell lines, silver nanowires did not show acute toxicity. A significant decrease in cell viability was observed only after prolonged treatment, as previously described [41]. This toxicity could be reduced if the silver nanowires are formulated as part of a device, such as a wound healing dressing or a bone regeneration scaffold. This was shown by Verma et al., who found that silver nanowire toxicity was greatly reduced when not tested as free wires but as part of a thin film [41]. No evidence of membrane damage was observed for any of the cell lines tested. Gliga et al. previously observed that LDH release from human lung cells (BEAS-2) depended on the dimensions of the AgNPs. Testing particles of 10, 40 and 75 nm, they found no significant LDH release for the NPs ≥ 40 nm diameter [42]. Thus, we hypothesize that AgNWs may cause less membrane damage than other AgNPs due to their size. In conclusion, we did not observe any advantage of the use of carbon nanotubes to form AgNW composites. We confirmed the potential use of AgNWs as slow release reservoirs of silver ions. The use of silver nanowires for biomedical applications could be advantageous in applications where a slow and sustained release is required, and embedding of AgNWs in delivery platforms such as wound healing bandages can be used as a strategy to reduce potential cytotoxicity.

## 4. Materials and Methods

### 4.1. Materials

2′,7′-Dichlorofluorescin diacetate (≥97%), acetone, agar, chitosan from shrimp shells with low viscosity (degree of deacetylation ~85%, calculated by H^1^-NMR), dimethyl sulfoxide anhydrous, (≥99.9%), glutaraldehyde solution, hexamethyldisilazane (≥99%), sodium chloride, octaldehyde (99%), poly(vinylpyrrolidone) powder (55 kDa), phosphate-buffered saline tablets, a silver standard for AAS, sodium cacodylate trihydrate, trypsin-EDTA 0.25% solution, tryptone enzymatic digest from casein, Triton^™^ X-100 and yeast extract for microbiology were purchased from Sigma–Aldrich (Irvine, UK). Ethanol, glycerol (99%), hydrochloric acid (37%), isopropanol, L-(+)-lactic acid (90 %) and sodium borohydride were purchased from Acros Organics (Geel, Belgium). 3-(4, 5-dimetheylthiazol-2)-2, 5 diphenyl tetrazolium bromide (MTT), 4’,6-diamidino-2-phenylindole dihydrochloride (DAPI), dimethylformamide, DMEM (high glucose, with GlutaMAX^TM^ and pyruvate), fetal bovine serum (FBS), Hank’s Balanced Salt Solution (HBSS), HPLC-grade water, methanol, nitric acid (70%), penicillin/streptomycin solution, phalloidin dylight 550, Pierce ™ BCA Protein Assay and silver nitrate were purchased from Fisher (Loughborough, UK). Single-wall carbon nanotubes-COOH OD 1–4 nm (SW-CNTs) were purchased from Cheaptubes.com (Grafton, VT). Corning DMEM/F12 (with L-glutamine and 15 mM HEPES) was purchased from Scientific Laboratory Supplies (UK).

### 4.2. Synthesis and Characterization of AgNWs

#### 4.2.1. Synthesis of AgNWs and Composites

AgNWs were synthesized via the polyol method, as previously described [43]. AgNWs-CNT were synthetized in the presence of CNTs with a slightly modified method. After complete dissolution of PVP in glycerol, 5.8 mg of carbon nanotubes was added and sonicated for 2 h to favor dispersion. After sonication, the synthetic procedure was the same as that used for AgNWs. The reaction progress was monitored by UV-Vis in the wavelength range of 300–600 nm (Thermo Scientific Nicolet Evolution 100 UV-Visible Spectrophotometer, Loughborough, UK). As a control, a physical mixture of CNTs and AgNWs (AgNWs-CNT mix) was prepared. Briefly, *N*-octyl-*O*-sulphate chitosan (NOSC, 10 mg, synthetized as previously described [44]) was dissolved in 10 mL of purified water before the addition of 12.5 mg of CNTs. The sample was sonicated for 4 h. Then, 40 mL of deoxygenated water and 2 g of PVP were added to the mixture. The mixture was then heated and maintained at 80 °C for 30 min. AgNWs in deoxygenated water (10 mL, 1.5 mg/mL) were then added to the reaction mixture; this was stirred at 800 rpm for 6 h in the dark. The sample was then centrifuged at 2880 g for twenty minutes and the supernatant removed. This step was repeated twice. The solid pellet obtained was then washed and stored as previously described [43].

#### 4.2.2. Characterization of AgNWs and Composites

Freeze-dried samples were suspended in deionized water at a concentration of 0.1 mg/mL; suspensions were dropped onto TEM grids (Agar scientific square mesh TEM support grids—copper) and allowed to dry at room temperature and pressure; no further treatment was applied. Dry grids were stored in sealed Petri dishes in a desiccator until analysis with an FEI CM120 BioTwin transmission electron microscope. The crystal structure of the samples was determined with a Bruker D2-Phaser diffractometer. Instrumental parameters were as follows: CuKα radiation, 30kV, 10 mA, LynxEye PSD detector with an angular opening of 5°, 2θ range 10°–80°, step size 0.020°, time per step 1 s, spinner 7.5 rpm. The alignment of the instrument was calibrated using an international standard (NIST 1976b). A low-background silicon crystal specimen holder (Bruker) was used. The analyses were performed at 26 ± 2°C. The XRD patterns were evaluated using Bruker EVA 14.2 (DIFFRAC^plus^ Package) software coupled with the database PDF-2 (ICDD). The total silver content and silver release were determined after digesting the freeze-dried samples in an equal volume of MilliQ water and 70% nitric acid. The volume of digested samples was brought up to 20 mL with MilliQ water. Finally, the solutions containing CNTs were filtered five times by a glass microfiber filter (GF/D). The silver content was determined by an inductively coupled plasma optical emission (ICP-OES) spectrometer (Spectroblue OEP-TI, Ametek, Germany) equipped with an ASX-520 autosampler. External calibration was performed by analysis of a blank and five solutions of dissolved AgNO_3_ standard in 2% HNO_3_ ranging from 0 to 100 μg L^−1^. The charged Ag ions were measured at two wavelengths (328.068 and 328.289 nm), and the results were averaged. The ICP-OES was equipped with Spectro Smart Analyzer software (vs. 6.01.0943). The set parameters for the analysis were as follows: 1450 W for plasma power, 30 rpm for pump speed, 13 L/min for coolant flow and 0.75 L/min for nebulizer flow. Ag^+^ release from nanoparticles was determined as previously reported [43]. In order to determine whether either AgNWs or CNTs possessed any autofluorescence, freeze-dried NPs were suspended in deionized water and sonicated to provide a homogeneous suspension. Samples were excited at different wavelengths (390, 488, 550, 570, 633 and 670 nm), and the respective emission spectra were recorded. Images of the suspensions were then acquired by fluorescence microscopy (Zeiss Axio Imager Z1). Surface zeta potentials were measured using a Malvern Zetasizer (Nano ZS, Malvern, UK). Nanoparticles were suspended by sonication in deionized water. On average, twelve measurements per samples were carried out. The selected refractive index for silver nanoparticles was 1.333.

### 4.3. Antibacterial Activity

#### 4.3.1. Bacterial Culture Preparation

The antibacterial activity of AgNWs was examined by a suspension assay against gram-negative *E.coli (ATCC 25922)* and gram-positive *S. aureus (ATCC 25923),* methicillin-resistant *S. aureus (ATCC 12403) and S. saprophyticus (ATCC 15305).* The bacteria were transferred from −80 °C (30 % glycerol) into 5 mL of fresh sterile LB by a sterile toothpick and incubated (at 37 °C and 200 rpm) until the bacterial suspension was cloudy (1 day for *E.coli* and *S. aureus*, 2 days for MRSA and 3 days for *S. saprophyticus*) (MaxQ™ 8000, Thermo Scientific). Then, 50 µL of the bacterial suspension was transferred into 5 mL of fresh sterile LB, and the bacteria were further incubated at 37 °C until the suspension was newly cloudy (1 day for *E. coli*, *S. aureus* and *MRSA* and 2 days for *S. saprophyticus*).

#### 4.3.2. Preparation of Stock Solutions of Antibacterial/Cytotoxic Agents

Stock suspensions (10 mg/mL) of NPs were prepared in deionized water and were then diluted in the appropriate medium. AgNO_3_ was dissolved in water in order to obtain an Ag^+^ concentration of 10 mg/mL. All suspensions and the silver nitrate solutions were sonicated for 2 h in order to obtained homogenous preparations.

#### 4.3.3. Determination of the Minimum Inhibitory Concentration (MIC) and Minimum Bactericidal Concentration (MBC)

Bacterial suspensions were prepared as described above. Samples were tested at a range of concentrations (10, 100, 200, 500, 750, 1000, 1500, 2000 and 3000 µg/mL) against bacterial suspensions (10^7^ CFU/mL) to a final volume of 150 µL in 96 well plates. Medium with only bacteria served as a negative control. Then, the plates were incubated at 37 °C for 24 h with gentle shaking (60 rpm). The absorbance was read at 600 nm using the appropriate blank suspensions/solutions. MIC was determined through spectrophotometry as the lowest concentration visibly inhibiting the bacterial growth. MBC was determined by transferring 25 µL samples from each well onto a LB agar plate. After overnight incubation (12 h), the total number of colonies appearing on the culture plate was assessed. The MBC was determined as the concentration at which there was no microbial growth. All experiments were performed in duplicate.

#### 4.3.4. Bacterial Growth Kinetics

The effect of the prepared samples on bacterial growth kinetics was assessed as previously described [43].

#### 4.3.5. Protein Leakage From Bacteria

Bacteria were grown as described above and then centrifuged (13,000 g for 5 min), and the pellets were suspended in sterile PBS (pH 7.4) to a final concentration of 0.6 CFU/mL in the presence of two concentrations of samples to be tested: 12.5 and 100 µg/mL. Bacteria were incubated at 37 °C and 200 rpm. After 1 and 24 h incubation, 1 mL of sample was taken from each test tube and centrifuged (13,000 g for 5 min). The supernatant was then stored at −20 °C until further analysis. The bicinchoninic assay (Pierce ™ BCA Protein Assay) was carried out according to the manufacturer’s instructions. Briefly, 25 μL samples were mixed with 200 μL of working solution. Sample absorbance was read at 562 nm after two hours of incubation at 37 °C. The experiment was carried out in duplicate.

#### 4.3.6. Extracellular ROS Production

To determine levels of ROS generated, a fluorescent probe was used; 2, 7- dichlorofluorescein diacetate (DCFH-DA) is converted to highly fluorescent 2, 7- dichlorofluorescein (DCF) in the presence of reactive oxygen species (H_2_O_2_, HO^•^ and ROO^•^). Initially, the DCFH-DA probe was suspended in DMSO at a 10 mM concentration before being diluted with LB medium to create a 100 μM working solution. After 24 h of incubation of bacteria with the test samples, 1 mL samples were removed and centrifuged at 1300 rpm for 1 min. The supernatant (40 μL) was removed and incubated with 60 μL of the fluorescent probe for 30 min in the dark at 37 °C. Using an excitation wavelength of 485 nm and an emission wavelength of 520 nm, the fluorescence of the samples was read on a bench top fluorimeter (Agilent Cary Eclipse).

### 4.4. Cytotoxicity Studies

#### 4.4.1. Cell Culture

Human fetal osteoblasts (hFOB 1.19), human foreskin fibroblasts (Hs27) and human colorectal adenocarcinoma cells (Caco-2) were purchased from ATCC^®^. hFOB were maintained at 37 °C in 5% CO_2_ in a flask in Dulbecco’s Modified Eagle’s Ham/F12 medium (with L-glutamine and 12 mM HEPES) containing 10% fetal bovine serum (FBS) and 1% penicillin/streptomycin (P/S). Hs27 were maintained under the same physical conditions but using DMEM (high glucose, with GlutaMAX^TM^ and pyruvate), 10% FBS and 1% P/S. Caco-2 cells were maintained at 37 °C in 5% CO_2_ in MEM containing 10% FBS, 1% P/S, 2 mM L-glutamine and 1% non-essential amino acids (NEAA). Cell lines were used between passage 4 and 12.

#### 4.4.2. Cell Viability

Cell viability was evaluated by the MTT [3-(4, 5-dimetheylthiazol-2)-2, 5 diphenyl tetrazolium bromide] colorimetric technique. Briefly, 5000 cells/ well were plated in a 96-well plate with 100 µL of complete medium and incubated overnight to permit cell attachment. Stock suspensions of test samples in complete medium were sonicated for 4 h at 40 Hz. Aliquots of initial AgNW suspensions were added to the cell medium to final concentrations of 0.1, 1, 10, 25, 50 and 100 μg/mL. After 24 h of incubation, 100 µL of treatments were added to each well containing cells and incubated for 2 and 24 h. At these time points, the treatments were removed and cells were treated with 100 µL of complete medium containing MTT (0.5 mg/mL). The cells were incubated for 4 h in a 5% CO_2_ incubator for reduction of MTT by metabolically active cells. The reagent was removed, and the purple formazan crystal inside the cells was solubilized with 100 µL of DMSO. The formazan content was quantified by spectrophotometry at a wavelength of 570 nm (SpectraMax^®^ i3x, Molecular Devices). DMSO served as a blank. The experiments were carried out at least in triplicate.

#### 4.4.3. Cell Membrane Integrity

To evaluate cell membrane integrity, the lactate dehydrogenase (LDH) leakage assay was performed. Cells were plated at 10,000 cells/well. Then, cells were treated with nanoparticles in complete medium containing 2% FBS (as the serum may interfere with the assay). Spontaneous activity and maximum LDH activity were used as controls. The assay was carried out according to the manufacturer’s instructions (Pierce ™ LDH cytotoxicity assay). The absorbance was read at 490 and 680 nm. The absorbance read at 680 nm (background) was subtracted from the 490 nm absorbance before the calculation of LDH release.

#### 4.4.4. Intracellular ROS Production

Cells were plated in a sterile black 96-well plate at 25,000 cells/well in 100 µL of medium. Cells were incubated for 24 h; then, the medium was removed, and the cells were washed once with sterile HBSS containing Ca and Mg. Cells were further incubated with DCFH-DA 28 µM in HBSS for 45 min. After washing with HBSS, the cells were treated with the complete medium (2% FBS), containing the test samples, for desired periods of time. Fluorescence was measured as described in Section 4.3.6.

#### 4.4.5. Silver Uptake by Cells

Cells were plated at 30,000 cells/well (1 mL medium) in a 24-well plate and incubated for 24 h, as described above. Cells were exposed to nanoparticles at different concentrations (1 mL). After 24 h of exposure, the medium was removed, and the cells were gently washed twice with cold PBS to remove loosely attached Ag ions and/or NPs from the cell membrane. To each well, HPLC grade water (600 µL) was added, and then 70% HNO_3_ (600 µL). The nanoparticles were then digested for 20 min, and the solutions were brought to 21 mL with HPLC grade water. Controls were made with just cells. The silver content was determined by an inductively coupled plasma optical emission (ICP-OES) spectrometer (Spectroblue OEP-TI, Ametek, Germany) equipped with an ASX-520 autosampler. External calibration was performed by analysis of a blank and five solutions of dissolved Ag in 2% HNO3 ranging from 0 to 100 μg L^−1^. The charged Ag ions were measured at two wavelengths (328.068 and 328.289 nm), and the results were averaged. The ICP-OES was equipped with SPECTRO SMART ANALYZER software (vs. 6.01.0943). The set parameters for the analysis were 1450 W for plasma power, 30 rpm for pump speed, 13.00 l/min for coolant flow and 0.75 l/min for nebulizer flow.

#### 4.4.6. Fluorescence Imaging of AgNW Uptake

Cells were seeded on coverslips at a cell density of 10000 cells/well in 24-well plates (300 µL of medium). After overnight incubation at 37 °C and 5% CO_2_, cells were treated with AgNWs. At scheduled time points, cells on coverslips were fixed directly with paraformaldehyde (4%) in PBS (pH 7.4) for 15 min, washed with PBS, permeabilized for 10 min in 0.1% Triton X-100/PBS, washed twice with PBS, blocked with 2% bovine serum albumin (BSA) in PBS for 1 h, washed twice with PBS, stained with Phalloidin Dylight 550 in PBS (2 units/mL, stock solution 300 units/mL in methanol) for 1 h (300 µL, at room temperature), washed twice with PBS, stained with DAPI 2 µg/mL in PBS for 10 min and finally rinsed again with PBS. Coverslips were mounted on glass slides using PermaFluor Aqueous Mounting Medium. Samples were kept protected from light until imaging. Photos were taken with a fluorescence microscope (Zeiss Axio Imager Z1) equipped with a Hamamatsu HR camera and a color AxioCam MRc camera. Images were processed by Volocity 6.3 software.

## Figures and Tables

**Table 1:** MIC and MBC values (mg/mL) for AgNWs, AgNWs-CNT, AgNWs-CNT-mix, AgNO_3_ and CNTs. Data are reported as the mean ± SD (*n* ≥ 3). One-way ANOVA results are reported in the table. The post hoc Tukey multiple comparisons test results are shown with *when compared to Ag+ and with ^$^when compared to AgNW-CNT-mix. *^,$^ *p* < 0.05; **^,$$^ *p* < 0.01, ***^,$$$^ *p* < 0.001 and ****^,$$$$^ *p* < 0.0001.

Microorganism	Material	MIC (mg/mL)	MBC (mg/mL)	MBC/MIC
*E. coli* Anova MIC p < 0.0001 MBC p < 0.0001	AgNWs	0.42 ± 0.26 *	1.16 ± 0.27 ^$$^	**2.8**
	AgNWs-CNT	0.53 ± 0.54 *^$$$^	1.38 ± 0.55 ***	**2.6**
	AgNWs-CNT-mix	0.49 ± 0.07 ***	0.99 ± 0.16 ***	**2.0**
	AgNO_3_	0.01 ± 0.00	0.17 ± 0.05	17
	CNTs	> 3.00	> 3.00	-
*S. aureus* Anova MIC p < 0.0001 MBC p < 0.05	AgNWs	0.36 ± 0.24 ^$$$^	1.55 ± 0.63	4.3
	AgNWs-CNT	0.28 ± 0.24 ^$$$^	1.05 ± 0.80	**2.9**
	AgNWs-CNT-mix	0.44 ± 0.12	0.77 ± 0.16**	**1.8**
	AgNO_3_	0.01 ± 0.00 ^$$$^	0.1 ± 0.00	10
	CNTs	> 3.00	> 3.00	-
MRSA Anova MIC p < 0.01 MBC p < 0.0001	AgNWs	1.07 ± 0.40 *	2.53 ± 0.53 ****	**2.4**
	AgNWs-CNT	0.85 ± 0.85 *	2.00 ± 0.44 ****^$$^	**2.4**
	AgNWs-CNT-mix	0.67 ± 0.14 *	> 3.00 ****	-
	AgNO_3_	0.01 ± 0.00	0.07 ± 0.05	7
	CNTs	> 3.00	> 3.00	-
*S. saprophyticus* Anova MIC p < 0.05 n.s.	AgNWs	0.25 ± 0.21	0.66 ± 0.42	**2.7**
	AgNWs-CNT	0.08 ± 0.00	0.72 ± 0.60	9
	AgNWs-CNT-mix	0.08 ± 0.06	0.46 ± 0.36	5.8
	AgNO_3_	0.01 ± 0.00	0.01 ± 0.00	**1**
	CNTs	> 3.00	> 3.00	-

## Supplementary Materials

Supplementary materials can be found at https://www.mdpi.com/1422-0067/21/7/2303/s1.

## Abbreviations

AgNWs	Silver nanowires
AgNWs-CNT	Silver nanowires prepared in the presence of carbon nanotubes
AgNWs-CNT-mix	Silver nanowire and carbon nanotube physical mixture
CNTs	Carbon nanotubes
ROS	Reactive oxygen species
