# Supplementary material for: Evaluation of Antibacterial and Cytotoxicity Properties of Silver Nanowires and Their Composites with Carbon Nanotubes for Biomedical Applications
Source: Int J Mol Sci. 2020 Mar 26;21(7):2303. doi: 10.3390/ijms21072303 (PMC7178261; doi:10.3390/ijms21072303)
Supplement: Supplementary file 1 [file ijms-21-02303-s001.pdf]

International Journal of
Molecular Sciences

MDPI

Article

Evaluation of antibacterial and cytotoxicity properties of silvernanowires and their composites with carbon nanotubes for biomedical applications.

Arianna De Mori[1], Richard S. Jones[1], Matteo Cretella[1], Guido Cerri[2], Roger R. Draheim[1], Eugen Barbu[1], Gianluca Tozzi[3], Marta Roldo[1]*

[1] School of Pharmacy and Biomedical Science, University of Portsmouth, St Michael's Building, White Swan Road, PO1 2DT, Portsmouth, UK.
[2] Department of Natural and Territorial Sciences, University of Sassari, Via Piandanna 4, 07100 Sassari, Italy.
[3] Zeiss Global Centre, School of Engineering, University of Portsmouth, Anglesea Building, Anglesea Road, PO1 3DJ Portsmouth, UK.
* Correspondence: marta.roldo@port.ac.uk;

Received: date; Accepted: date; Published: date

Supplementary information

1. *SEM images of silver nanowires*

Different concentrations of freeze dried AgNWs, AgNWs-CNT and AgNWs-CNT-mix (0.1, 1, 1.5 mg/ml) were suspended in a 1 %w/v chitosan solution in lactic acid 0.1 M. The samples were vortex mixed for approximately 2 minutes, followed by sonication for a further 90 minutes. Each sample was then added drop wise on to SEM stubs. The samples were allowed to dry, under vacuum, for 48 hours, before being coated with gold, using a sputter coater (Polaron e500, Quoram Technologies, UK) and analyzed via SEM imaging (JEOL JSM-6060 LV Scanning Electron Microscope, UK). The images obtained are shown in Fig. S1.

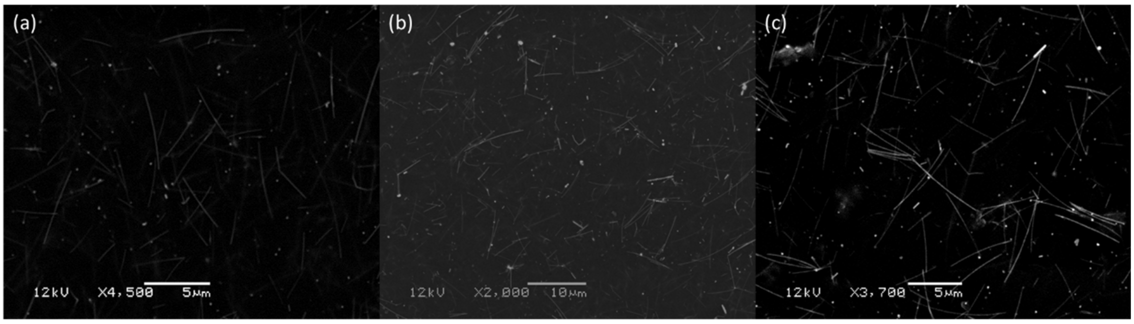

Figure S1. SEM images of: (a) AgNWs; (b) AgNWs-CNTs and (c) AgNWs-CNT-mix.

SEM images were analysed using ImageJ, each full wire present in the images was measured 3 times to determine the diameter and the length and size distribution graphs were obtained (Fig S2).

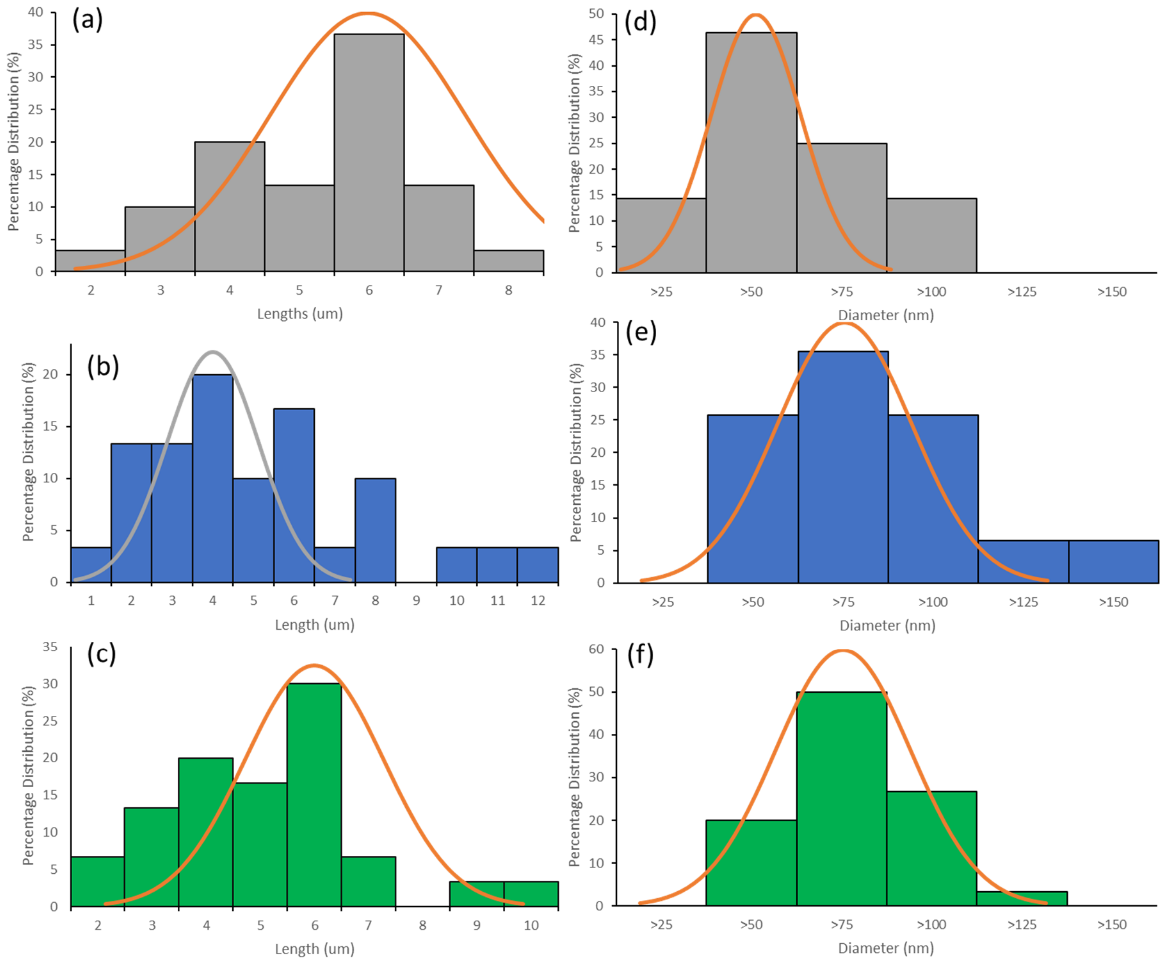

Figure S2. Distribution of the lengths and diameters of (a and d) AgNWs (b and e) AgNWs-CNT and (c and f) AgNW-CNT-Mix. Dimensions calculated from SEM image analysis using ImageJ software.

2. *Effect of CNTs and AgNWs-CNT-mix on bacterial growth curves*

CNTs and AgNWs-CNT-mix were used as control samples. CNTs (Fig. S3) did not have any effect on the growth of bacteria, while AgNWs-CNT-mix had some effect only at the highest concentration tested (Fig. S4)

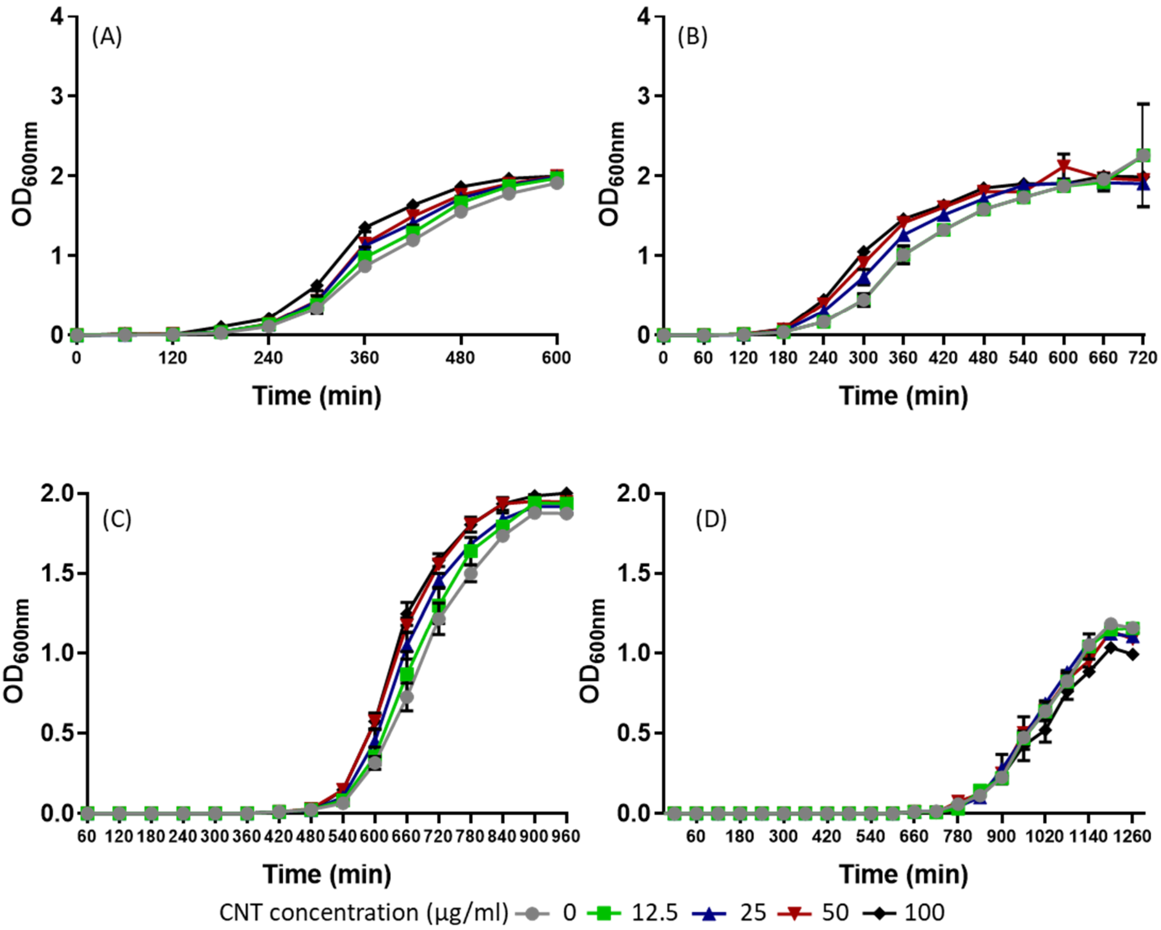

Figure S3. Growth curves of (A) *E. coli*, (B) *S. aureus*, (C) MRSA and (D) *S. saprophyticus* with different concentrations of CNTs. Results are reported as a mean ± SD (n=3).

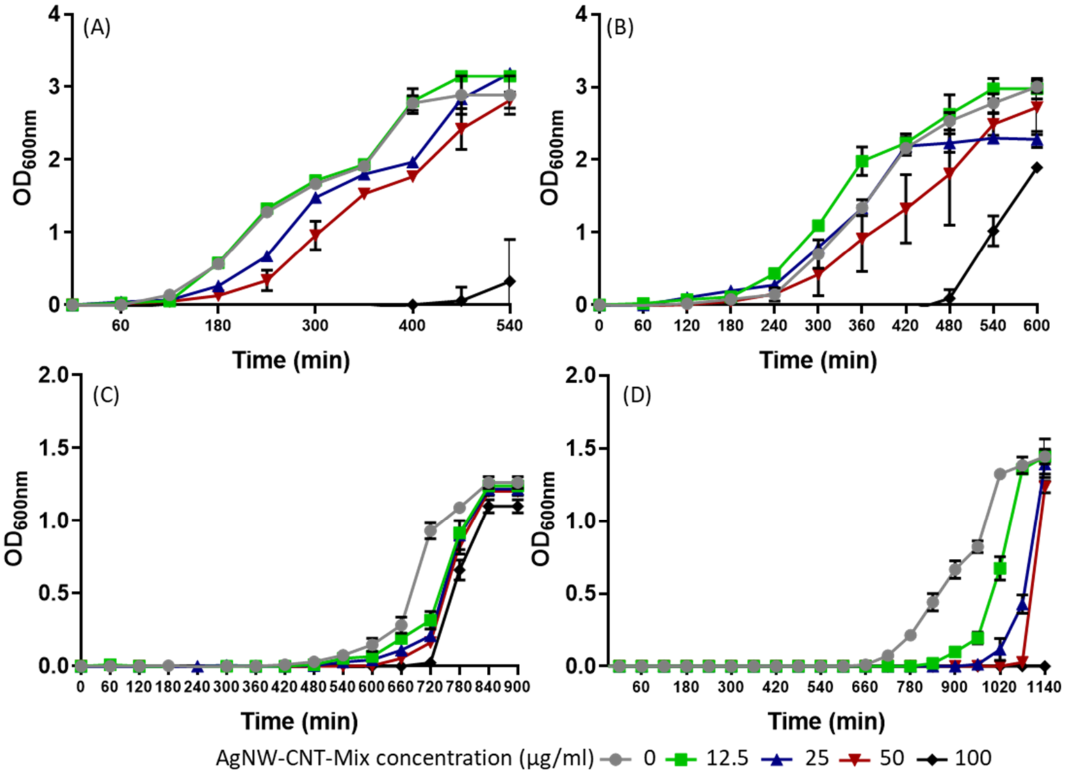

Figure S4. Growth curves of (A) *E. coli*, (B) *S. aureus*, (C) MRSA and (D) *S. saprophyticus* with different concentrations of AgNWs-CNT-mix. Results are reported as a mean ± SD (n=3).

3. *Effect of silvernanowires on lag time, rate of growth and stationary phase of bacteria*

Bacterial growth curves were analyzed to determine the effect of silver nanowires on the lag time, rate of growth and maxium bacterial concentration at the stationary phase (Fig. S5).

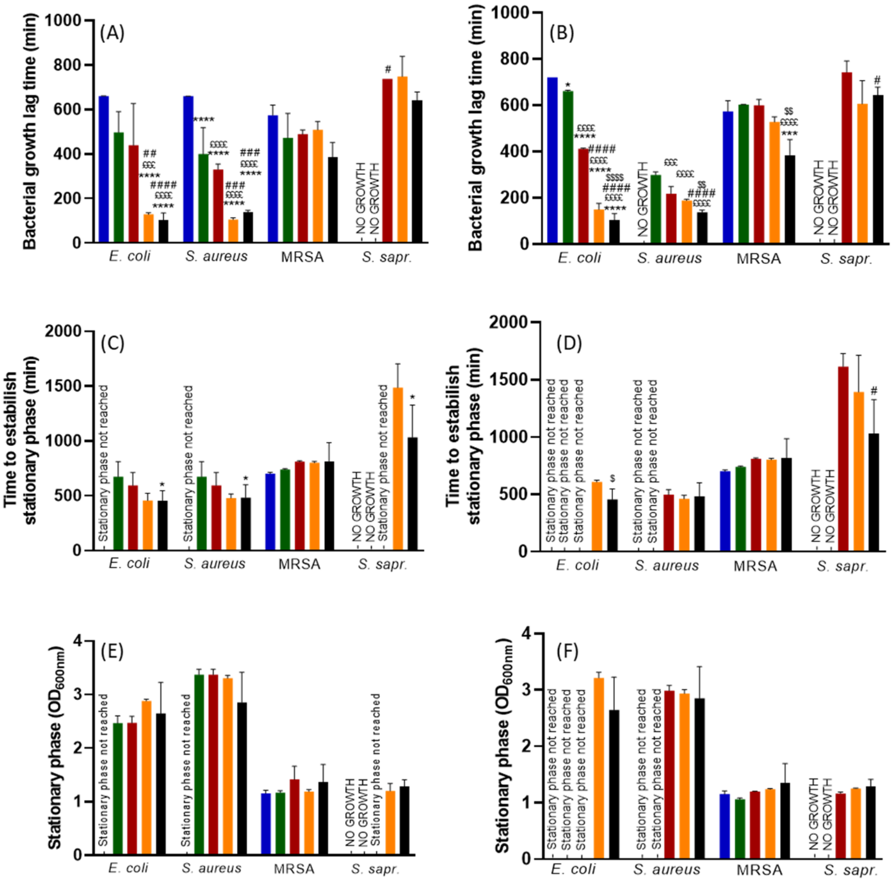

Figure. S5. Bacterial growth lag time (A, B); time to establish stationary phase (C, D) and max absorbance at stationary phase (E, F) of bacterial cultures treated with different concentrations (blue 100; green 50; red 25; yellow 12.5 and black 0 µg/ml) of AgNWs (A, C, E) and AgNW-CNTs (B, D, F). Data are reported as average ± S. D. (n=3). One way Anova was performed for all parameters for each bacteria, Tukey post-hoc test was also performed, symbols indicate significant difference £compared to control; #compared to 25; *compared to 50; $ compared to 100 µg/ml

4. *Confocal imaging of bacterial cells treated with silver nanowires*

E. coli cultures were treated for 6 h with either AgNWs or AgNWs-CNTs (25 µg/ml), and then stained with the Live/Dead BacLightTM staining kit (Molecular ProbesTM, Life technologies), according to the manufacturer's instructions. Briefly, bacteria pellets were obtained by centrifugation, the supernatant was discarded and the pellet was resuspended in 1 ml of PBS (pH 7.4). Then, equal volumes (3 µl) of dye component A and B were added to 1 ml of bacterial suspension, and incubated in the dark at room temperature for 15 minutes. Finally, 5 µl of the stained bacterial suspension was placed on a microscope slide and covered with a coverslip; images were acquired with a fluorescence microscope (EVOS fl, AMG, Westover Scientific, Inc.). The fluorescence images of the control showed numerous viable green colored *E. coli* cells. On the contrary, cells treated with AgNWs showed in proportion more red staining. Moreover, as already shown by growth curves analysis, AgNWs appeared to interfere with the bacterial reproduction, leading to a decreased number of cells in the samples.

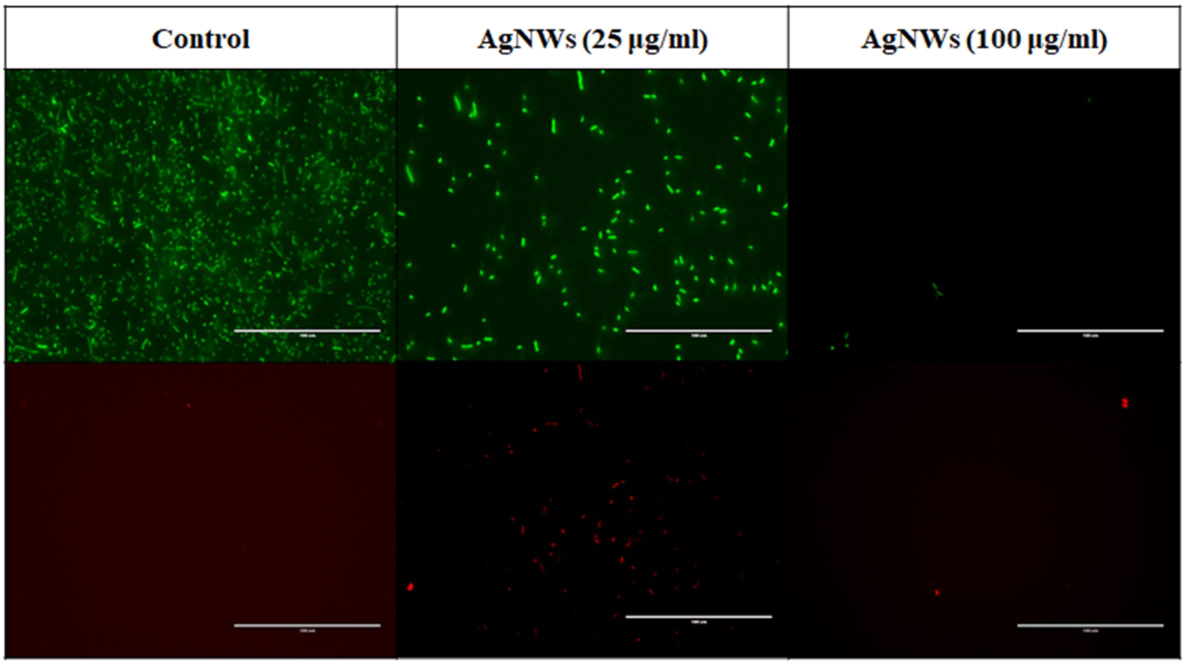

Fig. S6 Live/dead staining of *E. coli* treated with different concentrations of AgNWs (0, 25 and 100 µg/ml) for 6 hours. Scale bar 100 µm.

5. *ROS generation from bacteria*

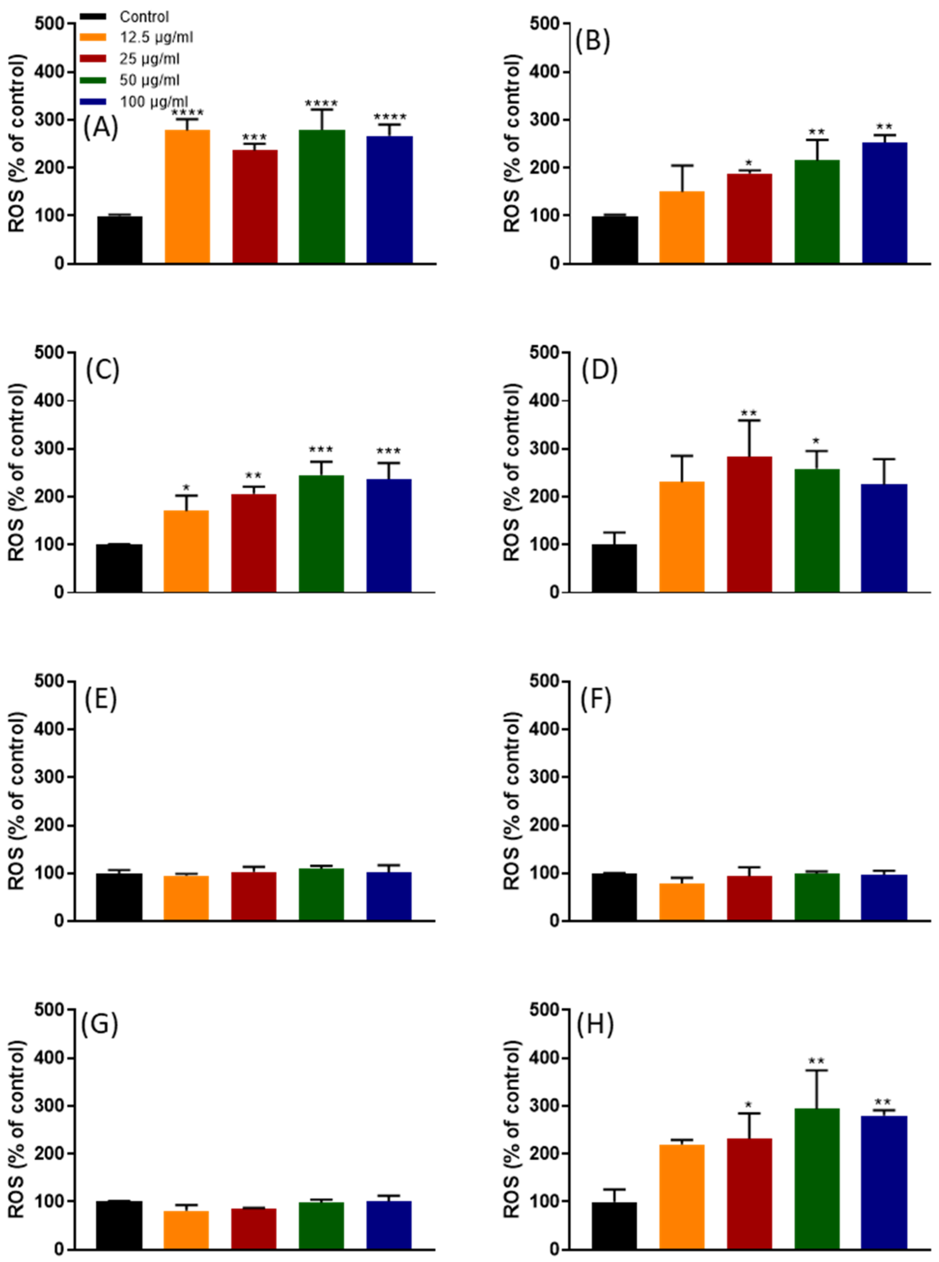

Figure S7. ROS production (% of the control) from bacterial cells, 24 h from treatment. *E. coli* (A and B), *S. aureus* (C and D), MRSA (E and F) and *S. saprophyticus* (G and H) treated with AgNWs (A, C, E, G) or AgNWs-CNT (B, D, E, F). Data are represented as a mean ± SD (n=3). The One-way ANOVA performed on all the samples showed significantly different ROS production for both AgNWs and AgNWs-CNT ($p<0.05$) for some of the bacterial strains. Results of the post-hoc Tukey's multicomparison test are shown in the graphs (* indicates $p < 0.05$, ** indicates $p < 0.01$, *** indicates $p < 0.001$ and **** indicates $p < 0.0001$)

5. Morphological analysis in bacteria treated with AgNWs and AgNWs-CNT by SEM imaging

Bacterial suspensions were prepared as described in 4.3.1. Then, bacteria were diluted to an OD_{600} of 0.01 and they were treated with different concentrations of nanoparticles (50 and 800 μg/ml) and incubated for 8 hours. The experiment was carried out in 96 well plates (150 μl).

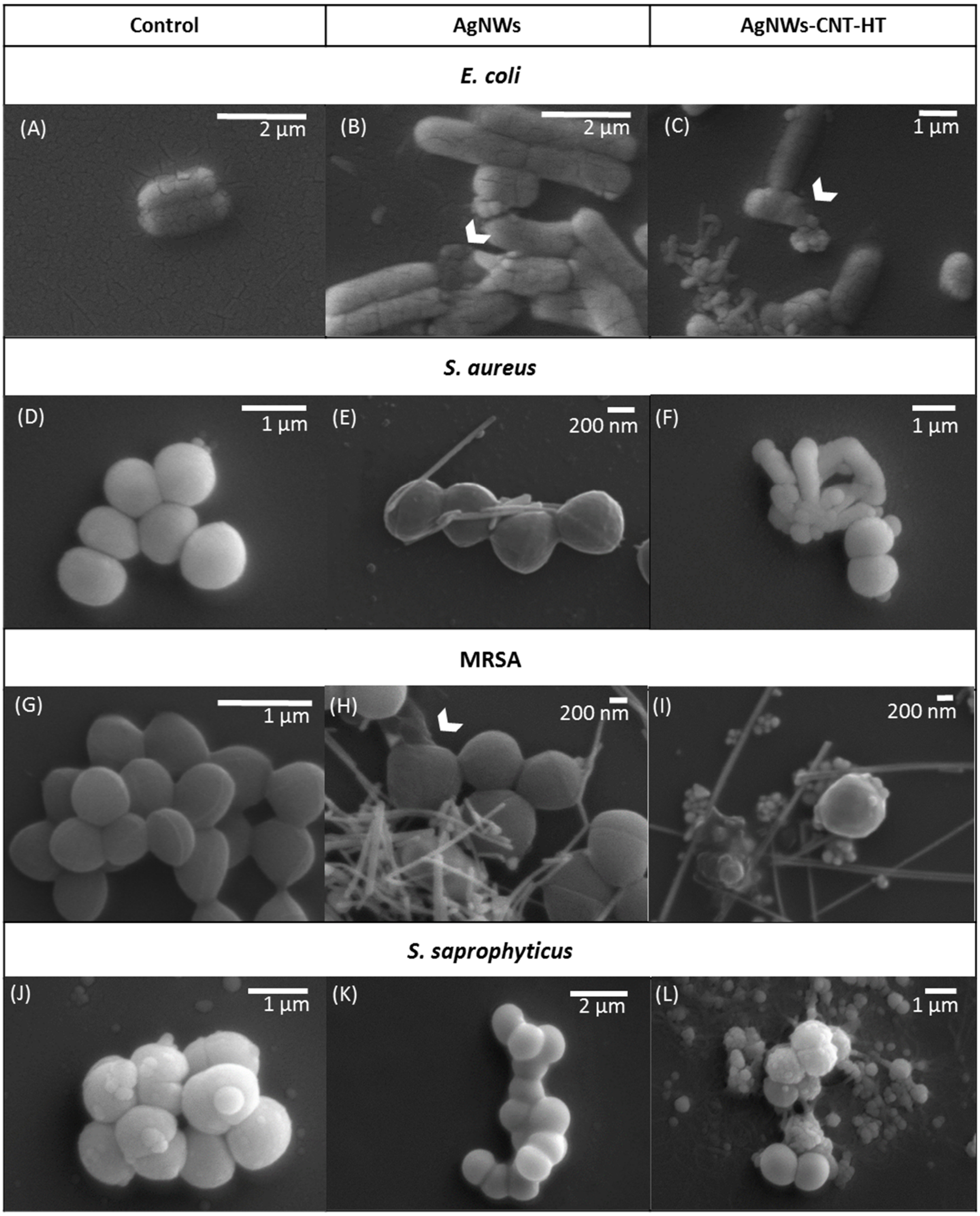

Figure S8. SEM photos of bacteria morphology after treatment either with AgNWs or AgNWs-CNT. (A), (B) and (C): *E. coli;* (D), (E) *and* (F): *S.aureus*, (G), (H) and (I): MRSA; (J), (K) and (L): *S. saprophyticus.*

The samples were then aseptically removed and centrifuged at 5000 x g for 10 min, the medium was removed and 800 μl of 0.2 M cacodylate was added to suspend the pellet. Then, 50 μl of cell suspension was added to a sterile silicon wafer (shiny side up) (Agar Scientific) and it was allowed

to settle for 40 min. Glutaraldehyde (0.5 ml, 2%) in 0.2 M cacodylate was gently added to each silicon wafer, making sure the specimens were completely immersed. The samples were fixed for 1 hour, at 4°C. Then, the samples were washed once with buffer and were dehydrated through a series of ethanol solutions (30, 50, 70, 90, 95 and 100%) for 5 min each at room temperature. Finally, samples were covered with acetone (until evaporation) and in 100% hexamethyldisilazane (HMSD) before being allowed to air-dry overnight. Images were taken by a scanning electron microscope (Zeiss EVO MA10), after having sputter coated the samples with gold and palladium (Polaron e500, Quoram Technologies, UK). Images of the 4 bacterial strains were acquired by SEM (Fig. S8) to detect potential morphological changes, 8h after treatment with AgNWs and AgNWs-CNT. AgNPs were noted to deposit on the surface of bacteria and with cell surface damage evident. The ability of AgNPs to cause big gaps formation in *E. coli* membrane has already been shown by Das et al. [1].

6. *ROS production by cells treated with CNTs*

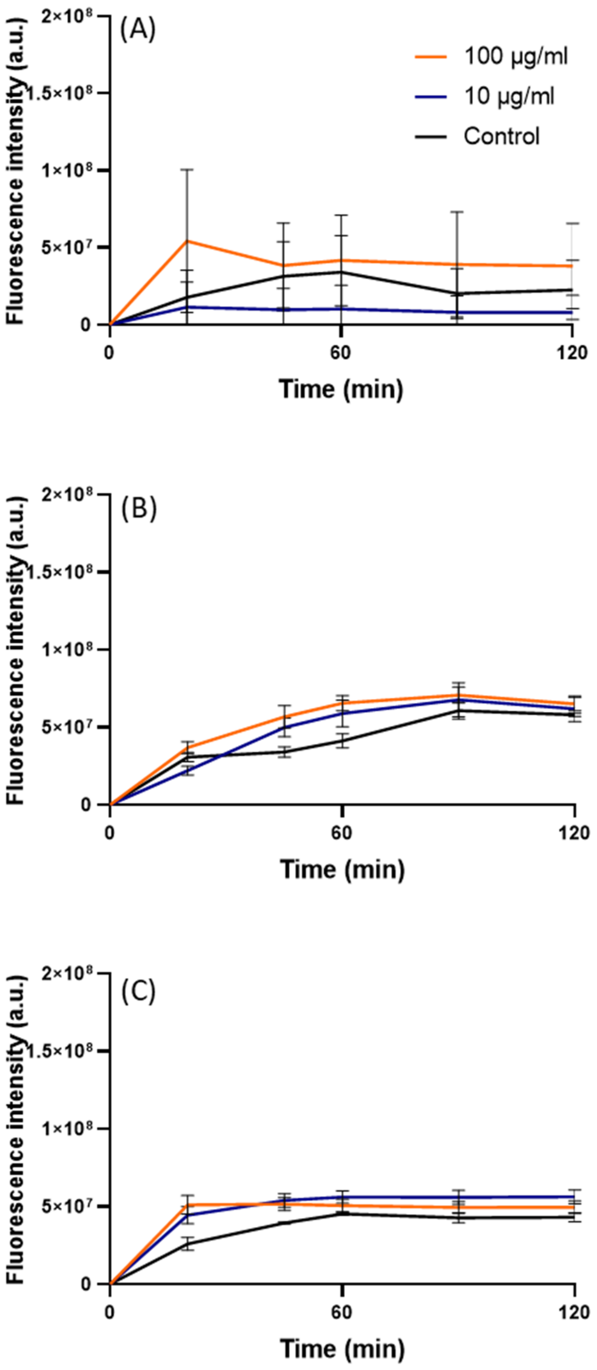

Figure S9. ROS production by CaCo2 (A); osteoblasts (B) and fibroblasts (C) treated with CNTs. Data are reported as mean ± S.D. (n=3).

7. Mitochondrial membrane potential

Mitochondrial membrane potential (ΔΨm) was measured using a mitochondrial permeability detection kit, containing JC-1 (tetraethylbenzimidazolylcarbocyanine iodide) (Abcam, Cambridge, UK). When the mitochondrial membrane (MM) is hyperpolarized, JC-1 enters the mitochondria and aggregates yielding a red colored emission (ca. 590 nm), whereas when the MM is depolarized, JC-1

is mainly a monomer that yields green fluorescence (ca. 529 nm). Briefly, 1,500 cells/well were plated in black 96 well plates and allowed to attach overnight. The day after, cells were treated with the nanomaterials for 24 h. Then, they were rinsed three times with PBS and stained with 20 μM JC-1 for 20 min at 37°C. Finally, the cells were washed twice with PBS and the fluorescence was measured (ex 475 nm; em 530 and 590 nm).

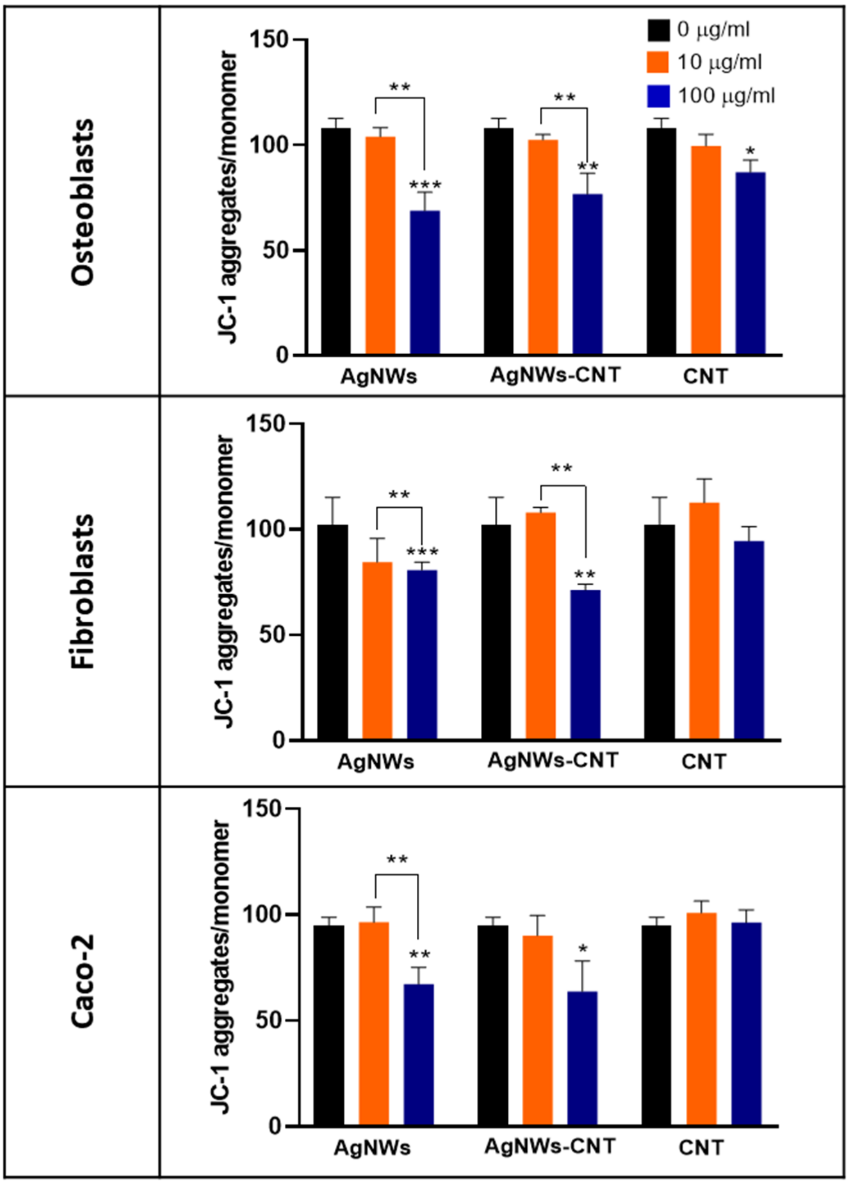

Figure S10. Mitochondrial membrane potential of osteoblasts, fibroblasts and Caco-2 cells treated with AgNWs, Ag-NWs-CNT and CNTs at different concentrations. Data are reported as mean ± SD (n=3). Data were analyzed by one-way Anova and Tukey's multicomparison test.

Loss of mitochondrial transmembrane potential ($\Delta\Psi_M$) is a significant signal of early phase apoptosis. Healthy mitochondria generate an electrochemical proton gradient across their membrane to produce ATP. On the other hand, apoptotic mitochondria present opened permeability pores and loss of electrochemical gradient. In our study, we have used a lipophilic dye, called JC-1, which is an

indicator of the MM's condition, generating a fluorescent green signal in normal cells and a red one in injured ones. As reported by Sivandzase et al. [2], the red/green fluorescence ratio of the dye in the mitochondria can interpreted as a direct assessment of the health of the mitochondria: the higher the red/green ratio, the healthier the cells and vice-versa. In this study, results indicate that both AgNWs and AgNWs-CNT incubation for 24 hours reduced the MMP for all the cell types in a dose-dependent manner. These findings are in good agreement with previous literature that has highlighted a connection between an increased ROS production (by silver nanowires, too) with mitochondrial membrane damage [3,4]. Therefore, the results suggest that the production of ROS by AgNWs could have triggered the formation of MM pores.

References

1. Das, B.; Dash, S.K.; Mandal, D.; Ghosh, T.; Chattopadhyay, S.; Tripathy, S.; Das, S.; Dey, S.K.; Das, D.; Roy, S. Green synthesized silver nanoparticles destroy multidrug resistant bacteria via reactive oxygen species mediated membrane damage. *Arab. J. Chem.* **2017**, *10*, 862–876.
2. Sivandzade, F.; Bhalerao, A.; Cucullo, L. Analysis of the Mitochondrial Membrane Potential Using the Cationic JC-1 Dye as a Sensitive Fluorescent Probe. *Bio-protocol* **2019**, *9*, e3128.
3. Gurunathan, S.; Qasim, M.; Park, C.; Yoo, H.; Kim, J.-H.; Hong, K. Cytotoxic Potential and Molecular Pathway Analysis of Silver Nanoparticles in Human Colon Cancer Cells HCT116. *Int. J. Mol. Sci.* **2018**, *19*, 2269.
4. Lehmann, S.G.; Toybou, D.; Pradas Del Real, A.-E.; Arndt, D.; Tagmount, A.; Viau, M.; Safi, M.; Pacureanu, A.; Cloetens, P.; Bohic, S.; et al. Crumpling of silver nanowires by endolysosomes strongly reduces toxicity. *Proc. Natl. Acad. Sci. U. S. A.* **2019**, *116*, 14893–14898.

© 2019 by the authors. Submitted for possible open access publication under the terms and conditions of the Creative Commons Attribution (CC BY) license (http://creativecommons.org/licenses/by/4.0/).
